# Hypothermia-induced dystonia and abnormal cerebellar activity in a mouse model with a single disease-mutation in the sodium-potassium pump

**DOI:** 10.1371/journal.pgen.1006763

**Published:** 2017-05-04

**Authors:** Toke Jost Isaksen, Lieke Kros, Natascia Vedovato, Thomas Hellesøe Holm, Ariel Vitenzon, David C. Gadsby, Kamran Khodakhah, Karin Lykke-Hartmann

**Affiliations:** 1 Department of Biomedicine, Aarhus University, Aarhus, Denmark; 2 Centre for Membrane Pumps in Cells and Disease-PUMPKIN, Danish National Research Foundation, Department of Molecular Biology and Genetics, Aarhus University, Aarhus C, Denmark; 3 Dominick P Purpura Department of Neuroscience, Albert Einstein College of Medicine, Bronx, New York, United States of America; 4 The Laboratory of Cardiac/Membrane Physiology, The Rockefeller University, New York, New York, United States of America; 5 Aarhus Institute of Advanced Studies (AIAS), Aarhus University, Aarhus C, Denmark; 6 Department of Clinical Medicine, Aarhus University, Aarhus, Denmark; Florey Institute of Neuroscience and Mental Health, AUSTRALIA

## Abstract

Mutations in the neuron-specific α_3_ isoform of the Na^+^/K^+^-ATPase are found in patients suffering from Rapid onset Dystonia Parkinsonism and Alternating Hemiplegia of Childhood, two closely related movement disorders. We show that mice harboring a heterozygous hot spot disease mutation, D801Y (α_3_^+/D801Y^), suffer abrupt hypothermia-induced dystonia identified by electromyographic recordings. Single-neuron *in vivo* recordings in awake α_3_^+/D801Y^ mice revealed irregular firing of Purkinje cells and their synaptic targets, the deep cerebellar nuclei neurons, which was further exacerbated during dystonia and evolved into abnormal high-frequency burst-like firing. Biophysically, we show that the D-to-Y mutation abolished pump-mediated Na^+^/K^+^ exchange, but allowed the pumps to bind Na^+^ and become phosphorylated. These findings implicate aberrant cerebellar activity in α_3_ isoform-related dystonia and add to the functional understanding of the scarce and severe mutations in the α_3_ isoform Na^+^/K^+^-ATPase.

## Author summary

The neurological spectrum associated with mutations in the *ATP1A3* gene, encoding the α_3_ isoform of the Na^+^/K^+^-ATPase, is complex and still poorly understood. To elucidate the disease-specific pathophysiology, we examined a mouse model harboring the mutation D801Y, which was originally found in a patient with Rapid onset Dystonia Parkinsonism, but recently, also in a patient with Alternating Hemiplegia of Childhood. We found that this model exhibited motor deficits and developed dystonia when exposed to a drop in body temperature. Cerebellar *in vivo* recordings in awake mice revealed irregular firing of Purkinje cells and their synaptic targets, the deep cerebellar nuclei neurons, which was further exacerbated and evolved into abnormal high-frequency burst firing during dystonia. The development of specific neurological features within the *ATP1A3* mutation spectrum, such as dystonia, are thought to reflect the functional consequences of each mutation, thus to investigate the consequence of the D801Y mutations we characterized mutated D-to-Y Na^+^/K^+^-ATPases expressed in *Xenopus* oocytes. These *in vitro* studies showed that the D-to-Y mutation abolishes pump-mediated Na^+^/K^+^ exchange, but still allows the pumps to bind Na^+^ and become phosphorylated, trapping them in conformations that instead support proton influx.

## Introduction

Dystonia is a movement disorder characterized by involuntary sustained or repetitive muscle contractions, causing twisting movements and abnormal postures [1, 2]. It is usually caused by head injuries, drug side effects, metabolic insult, or genetic alterations, and is thought to involve the neuroanatomic circuitry of the basal ganglia, sensorimotor cortex, brainstem, and cerebellum [3].

While most dystonias are idiopathic, some are familial, and modifications of more than 25 designated genes associated with dystonia (DYTs) have been described [4]. Several mutations in the *ATP1A3* (*DYT12*) gene, encoding the neuron-specific α_3_ isoform of the Na^+^/K^+^-ATPase, can cause rapid-onset dystonia-parkinsonism (RDP) characterized by an abrupt onset of dystonia and parkinsonian motor-related features [5], or alternating hemiplegia of childhood (AHC) characterized by fluctuating spells of tonic, dystonic, hemiplegic and oculomotor abnormalities [6–8]. A separate mutation in *ATP1A3* is responsible for the Cerebellar ataxia, Areflexia, Pes cavus, Optic atrophy, and Sensorineural hearing loss (CAPOS) syndrome [9]. All three *ATP1A3* disorders are typically triggered by environmental and/or physiological events such as physical exhaustion, temperature changes, emotional stress, or infections, pointing to a broad spectrum of often distinct, yet overlapping, neurological disorders [10].

Intriguingly, missense mutations that cause different amino acid substitutions at the same position in the *ATP1A3* gene can cause distinct diseases. A prominent example is amino acid position 801, where different mutations cause RDP or AHC (D801Y)[5, 11, 12] or AHC (D801N, D801E and D801V)[6, 13–16]. That position 801 is a hotspot for disease-causing mutations correlates with its crucial role in Na^+^/K^+^-ATPase pump function; the aspartate residue there is conserved in all Na^+^/K^+^-ATPase isoforms of all animal species, where it alternately coordinates both K^+^ ions [17, 18] and two of the three Na^+^ ions transported [19, 20], and is required for enclosure of the K^+^-ions [21].

In the present study we show that a mouse model with the D801Y disease-mutation (α_3_^+/D801Y^ mice) [22] displayed severe hypothermia-induced dystonia, which correlated with abnormal cerebellar neuronal activity *in vivo*. *In vitro* pump characterization revealed that D-to-Y mutant pumps failed to carry out Na^+^/K^+^ exchange, but retained the ability to bind Na^+^. These data thus provide a heretofore unknown link between hypothermia and dystonia that implicates aberrant cerebellar activity in α_3_ isoform-related dystonias and provides functional insight into the disease-causing effects of the underlying Na^+^/K^+^-ATPase dysfunction.

## Results

### Hypothermia induce convulsion-like movements and dystonia-like postures in α_3_^+/D801Y^ mice

**Fig 1:**
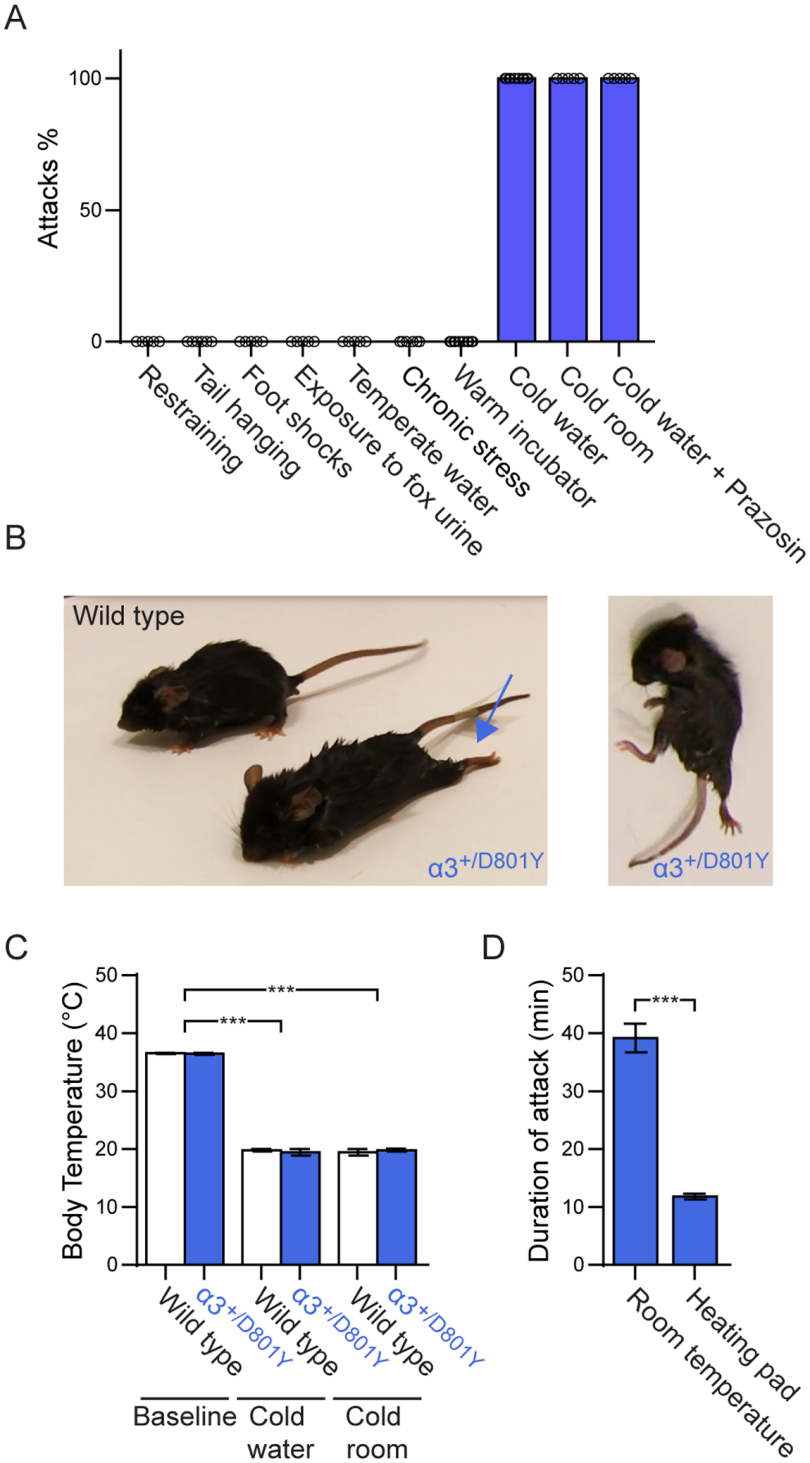
Hypothermic attacks. Hypothermia causes dystonia-like attacks in α_3_^+/D801Y^ mice. (A) Average occurrence (%) of an attack in α_3_^+/D801Y^ mice, following restraining for 10 min (n = 5), tail suspension for 6 min (n = 6), randomly timed electric foot shocks (n = 5), exposure to fox urine (n = 5), warm incubator (43°C) (n = 5), temperate water swim (35°C) (n = 6), chronic variable stress protocol (n = 11), cold water swim (5–10°C) (n = 10), cold environment (-20°C) (n = 6) and Prazosin treatment before cold water swim (n = 5). Only hypothermia, caused by cold water swim or cold environment exposure, consistently induced attacks in the α_3_^+/D801Y^ mice (n = 15 for cold water and n = 6 for cold environment). (B) Example of dystonic-like posture with hind limbs hyperextended caudally (left picture, arrow) and a period of convulsion with abnormal postures and twisting movements (right picture) in α_3_^+/D801Y^ mice after cold water swim. WT mice never displayed similar abnormal symptoms (left picture). (C) Core body temperature measured by rectal probe at onset of attack induced by exposure to cold water or cold environment. Both methods induced a significant drop in body temperature just below about 20°C before symptoms occurred in α_3_^+/D801Y^ mice. WT mice displayed identical drops in body temperature (n = 6 for both WT and α_3_^+/D801Y^). (D) Attack duration after induction by cold water when α_3_^+/D801Y^ mice were left to recuperate at room temperature or on a 33.3°C heating pad (n = 6).

As the abrupt onset of dystonia in both RDP and AHC patients usually occurs in response to a stressful environmental or physiological event [23], we subjected α_3_^+/D801Y^ mice to a variety of such conditions (Fig 1A). However, stress tests that included restraining, tail suspension, randomly timed electric foot shocks, exposure to fox urine, hyperthermia resulting in elevation of body temperature to 40.4 ± 0.3°C, and forced swimming in warm 35°C water did not result in genotype-specific abnormal symptoms. Even a 2-week chronic unpredictable stress protocol failed to provoke symptoms. In contrast, forced swimming in 5–10°C cold water for as little as 4 min consistently caused severe dystonia-like postures with hyperextended limbs (Fig 1B_left_), and long periods of body twisting and convulsion-like movements (Fig 1B_right_) in the α_3_^+/D801Y^ mice (Fig 1A and S1 Video). These symptoms were never observed in WT littermates (Fig 1B_left_ and Supplementary movie 1). The attacks were pulsating in severity and lasted on average 39.2 ± 2.5 min (n = 6), after which α_3_^+/D801Y^ mice fully recovered, with no apparent residual or persisting symptoms.

As a result of the 4 min cold-water swim, core body temperature dropped significantly to about 20°C in both α_3_^+/D801Y^ mice and WT littermates (α_3_^+/D801Y^: 36.5 ± 0.2°C to 19.5 ± 0.6°C (p<0.0001, two-way ANOVA with genotype (WT versus α_3_^+/D801Y^) and condition (baseline versus hypothermia) as main factors followed by Tukey's multiple comparisons test); WT: 36.5 ± 0.1°C to 19.8 ± 0.2°C (p<0.0001)) (Fig 1C) without any difference in either baseline or post swim body temperature between α_3_^+/D801Y^ and WT mice (p>0.9999 and p = 0.9873, respectively). Since only swimming in cold, and not in temperate water resulted in attacks, we speculated that lower body temperature, rather than the stress of forced swimming, was causative for the attacks. To address this, mice were placed in a freezing cold, -20°C, environment and kept there until attacks developed, or until core body temperature dropped below 20°C. Remarkably, all six α_3_^+/D801Y^ mice (but none of the six WT mice) developed identical attacks to those caused by the cold-water swim (Fig 1A) as their body temperature dropped below 20°C (Fig 1C), strongly indicating that lowered body temperature alone induced the attacks. To further rule out a stress aspect of the attacks, α_3_^+/D801Y^ mice were treated with the alpha-adrenergic blocker prazosin before being subjected to cold-water swim. All prazosin-treated α_3_^+/D801Y^ mice still developed attacks (Fig 1A) with durations and severity identical to those untreated.

If hypothermia indeed caused the attacks, rewarming the animal during an attack might be expected to reduce the duration and/or severity of the attack. Indeed, placing α_3_^+/D801Y^ mice on a 33.3°C heating pad immediately attack symptoms began, after induction by a cold-water swim, significantly diminished the duration of the attacks compared to the average time for recovery at room temperature (heating pad: 11.8 ± 0.5 min; room temperature: 39.2 ± 2.5 min (p = 0.0002, paired t-test)) (Fig 1D).

### Hypothermia-induced attacks are dystonic in nature

To explore the electrophysiological nature of these attacks, electrocorticographical (ECoG) recordings and hind limb electromyography (EMG) recordings were performed on five α_3_^+/D801Y^ mice before (baseline) and during hypothermia-induced attacks.

**Fig 2:**
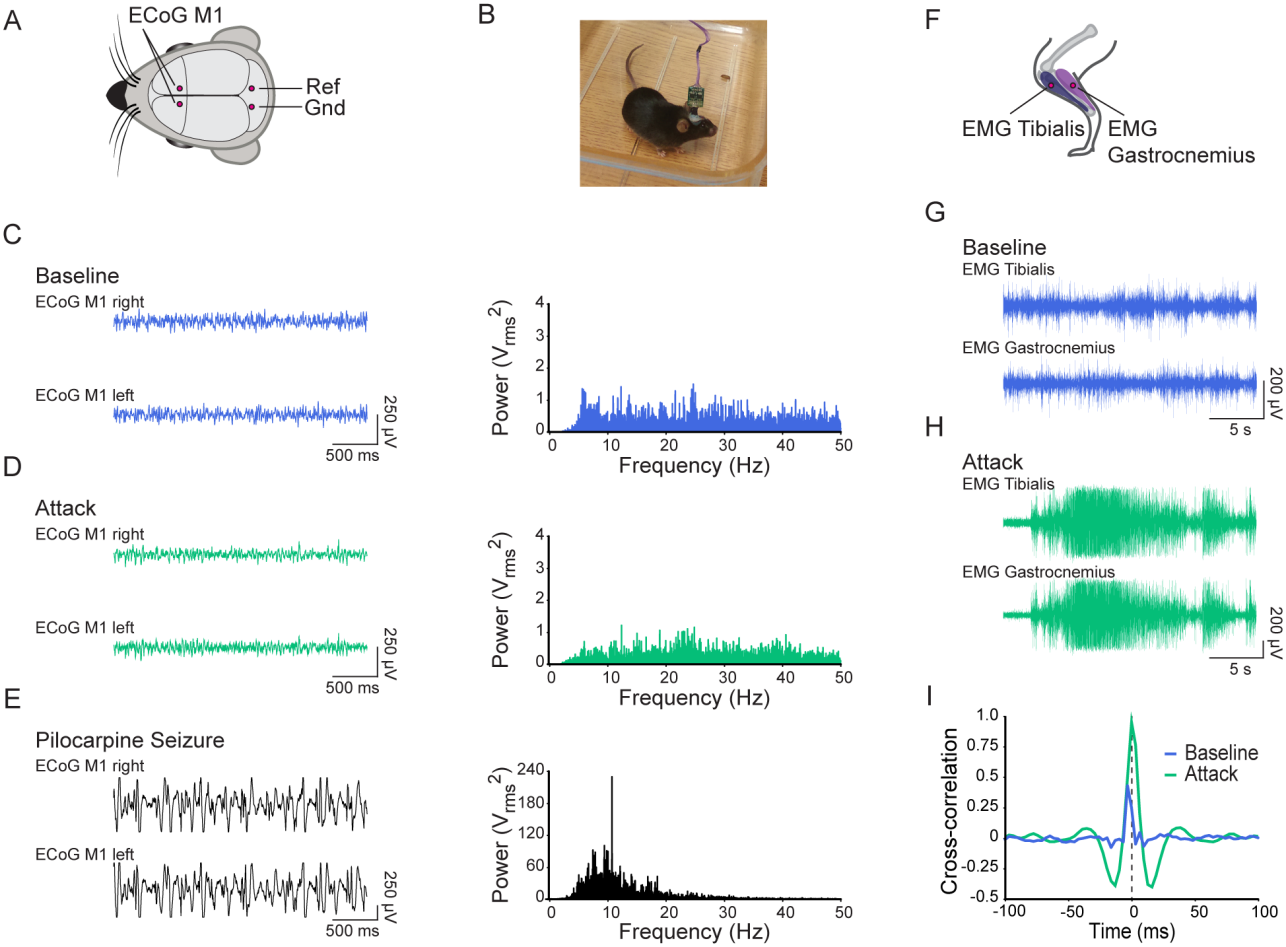
Dystonia. Hypothermia-induced attacks are dystonic of nature. (A) Illustration showing the locations of the ECoG electrodes. ECoG was bilaterally recorded from the primary motor cortex with ground and reference electrodes placed above the superior colliculi. (B) Picture of the experimental setting showing a α_3_^+/D801Y^ mouse freely moving in an empty cage while ECoG is recorded. (C) Representative example of ECoG (left) and corresponding power spectrum of a baseline measurement during which the mouse is exploring the cage. (D) As in C but the recording was made during an attack induced by cold water exposure in the same α_3_^+/D801Y^ mouse. (E) As in C and D but recorded during a pilocarpine induced tonic-clonic seizure in the same mouse (note the difference in y-axis of both the ECoG and power spectrum). (F) Illustration indicating locations of EMG recordings from the tibialis and gastrocnemius in the hind limb. (G, H) Representative examples of EMG recorded from the same α_3_^+/D801Y^ mouse from the anterior tibialis and gastrocnemius pre (B, blue) and post (C, green) a cold water induced attack. (I) Cross correlograms of the traces shown in G (blue) and H (green) showing a pronounced difference in correlation between activity of agonist and antagonist hind limb muscles indicative of dystonic postures during an attack.

ECoGs were recorded bilaterally from the primary motor cortex in freely moving animals (Fig 2A and 2B). None of the α_3_^+/D801Y^ mice showed any epileptic activity during the attacks, and there was no evidence of genotype-related differences in ECoG signals or power spectra between baseline recordings and recordings during attacks (Fig 2C and 2D, respectively). As an experimental control, seizures were induced using a lithium-pilocarpine protocol, which resulted in the expected dramatically different ECoG activity and corresponding power spectra (Fig 2E).

Simultaneous EMG recordings from the anterior tibialis and gastrocnemius muscles of the hind limb (Fig 2F) revealed a pronounced increase in co-contraction of these muscles during the attacks compared to baseline (Fig 2G and 2H), characteristic of dystonia. This was quantified with representative cross correlograms (Fig 2I). From the absence of epileptic activity in ECoG, and the evidence for co-contractions in the EMG recording, we conclude that the attacks were dystonic in nature.

### Motor deficits in α_3_^+/D801Y^ mice

**Fig 3:**
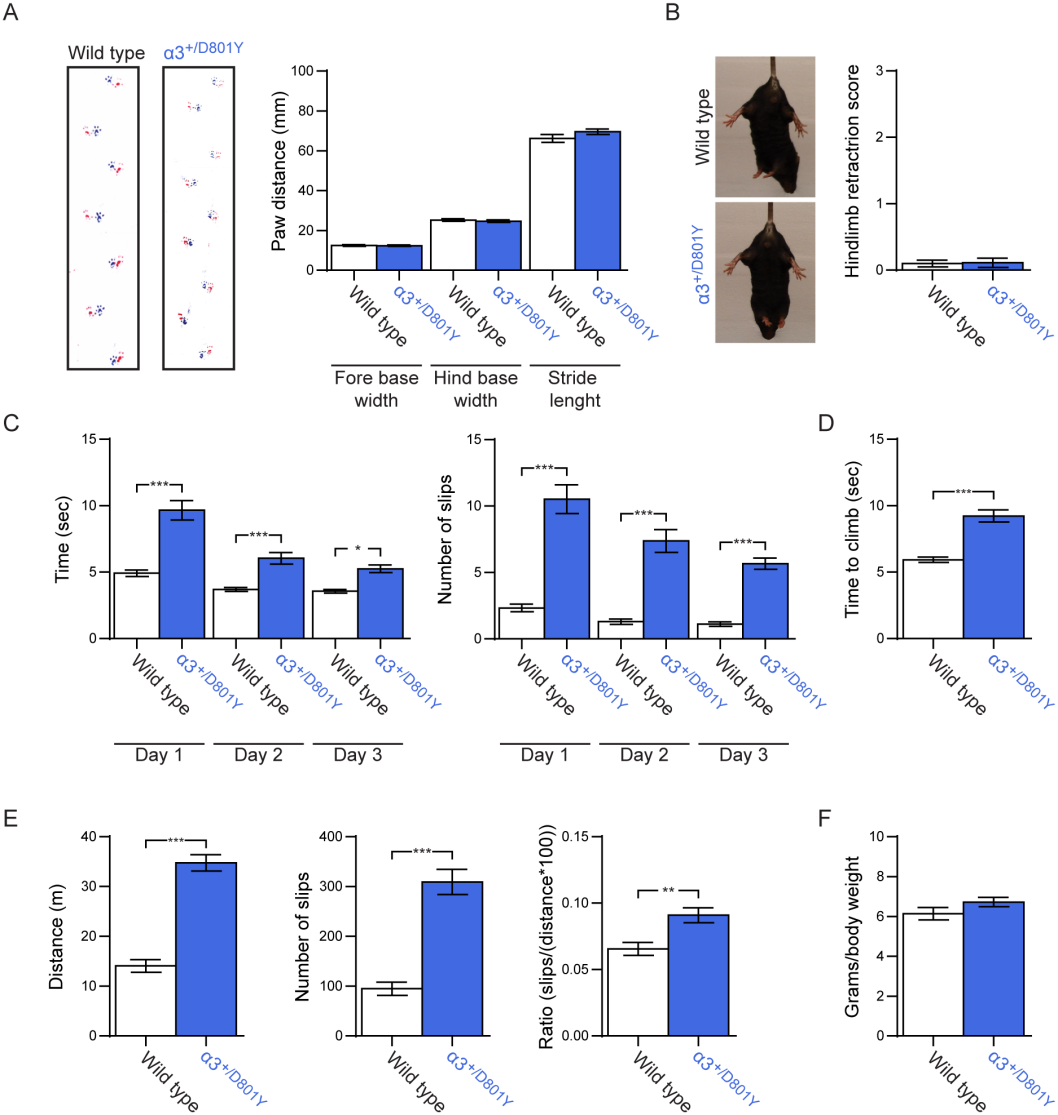
Ataxia. α_3_^+/D801Y^ mice display moderate motor deficits. (A) Gait analysis with fore and hind base width and stride length (n = 6 for both WT and α_3_^+/D801Y^). Front paws were colored blue, while hind paws were colored with red paint. (B) Hind limb clasping test (n = 10 for WT and n = 6 for α_3_^+/D801Y^). (C) Balance beam test over 3 consecutive days, with time to cross (left) and number of slips (right) (n = 24 for WT and n = 23 for α_3_^+/D801Y^). (D) Rope climb test with time to climb (n = 19 for WT and n = 23 α_3_^+/D801Y^). (E) Parallel rod floor test with distance traveled, number of slips and ataxia ratio defined by: number of slips/(distance*100) (n = 10 for WT and n = 12 for α_3_^+/D801Y^ mice). (F) Grip strength (n = 12 for WT and n = 13 for α_3_^+/D801Y^). All data shown are means ± SEM. *p<0.05, **p<0.01, ***p<0.001.

Besides dystonia, patients also suffer from ataxia and other motor-related features. Thus, α_3_^+/D801Y^ mice and WT littermates were subjected to motor tests. Although the α_3_^+/D801Y^ mice exhibited normal body posture, normal gait (p = 0.8919 fore base width, p = 0.5428 hind base width, and p = 0.1856 stride length, t-test) (Fig 3A), and an absence of hind limb clasping (p = 0.8981, t-test) (Fig 3B), we did find that α_3_^+/D801Y^ mice performed considerably worse than WT littermates in more challenging and stressful motor tests. On the balance beam, α_3_^+/D801Y^ mice needed more time to cross (p<0.0001 day 1, p = 0.0003 day 2, and p = 0.0335 day 3, two-way ANOVA with genotype (WT versus α_3_^+/D801Y^) and time (days) as main factors followed by Tukey's multiple comparisons test), and had more foot slips (p<0.0001 day 1, p<0.0001 day 2, and p<0.0001 day 3) when compared to WT littermates over three consecutive days (Fig 3C) (Supplementary movie 2). The α_3_^+/D801Y^ mice also took significantly more time to climb a vertical rope than WT littermates (p<0.0001, t-test) (Fig 3D), and they exhibited a higher ataxia ratio in the parallel rod floor test (p = 0.0032, t-test) (Fig 3E), but their grip strength was comparable to that of WT littermates (p = 0.1358, t-test) (Fig 3F).

### The α_3_ isoform in cerebellum

Purkinje cells are thought to express solely the α_3_ isoform, and not the otherwise ubiquitously-expressed α_1_ isoform [24, 25], and are therefore suggested to be particular highly sensitive to modifications of α_3_ isoform function [26, 27]. We therefore investigated whether aberrant cerebellar function could be the cause of the observed motor deficits and inducible dystonia.

**Fig 4:**
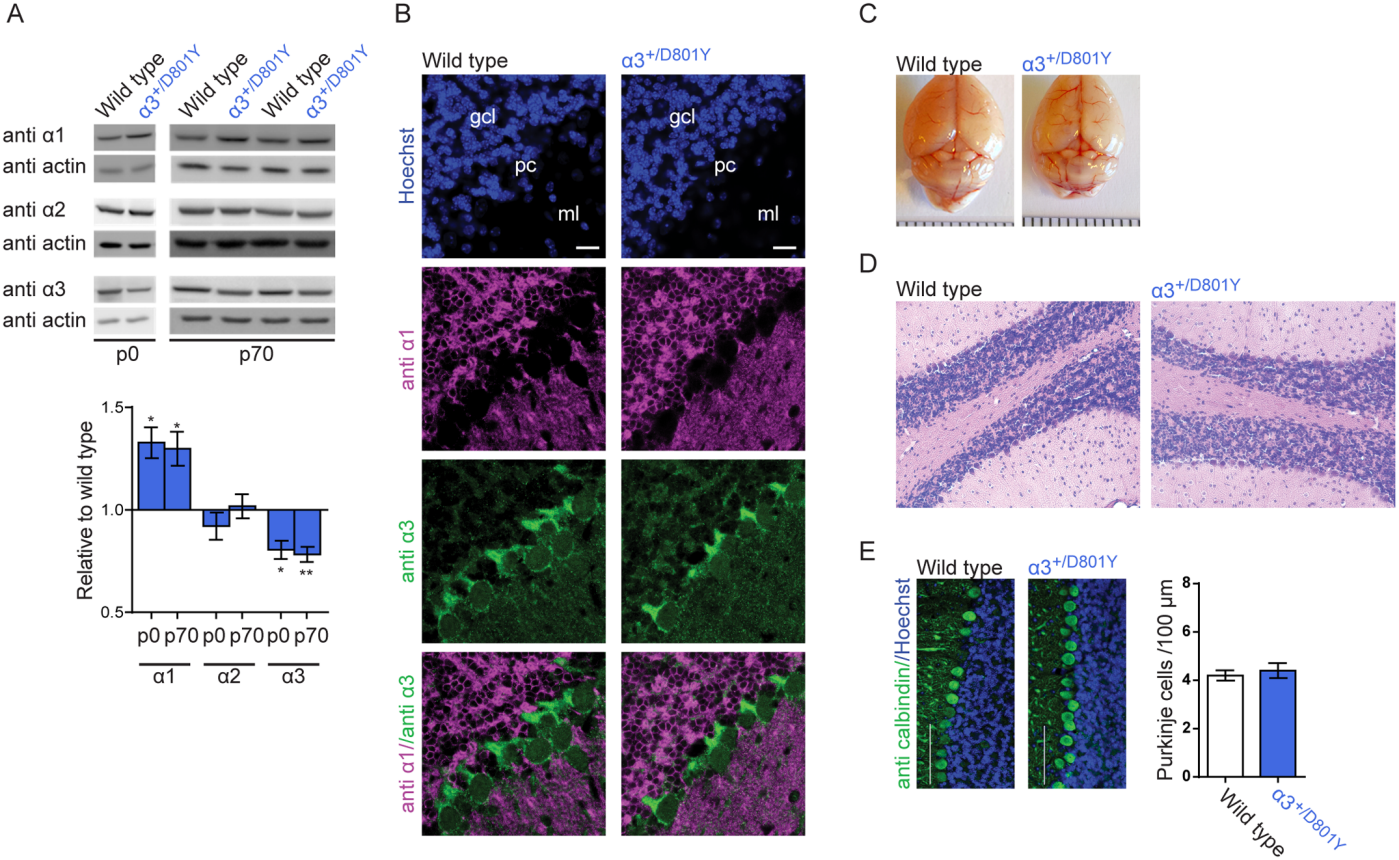
α_3_ in cerebellum. Na^+^/K^+^-ATPase expression and gross cerebellar morphology in α_3_^+/D801Y^ mice. (A) Western blot of cerebellar lysates from p0 and p70 α_3_^+/D801Y^ mice and WT littermates with antibodies against α_1_, α_2_ and α_3_ Na^+^/K^+^-ATPase isoform and actin as loading control. Quantification of blots is presented below as expression relative to WT (n = 6 for each group). Full-length Western blots are shown in Supplementary S1 Fig. (B) Immunofluorescence staining of cerebellum from WT and α_3_^+/D801Y^ mice using antibodies against the α_1_ (magenta) and α_3_ (green) isoform, with Hoechst (blue) for nuclear stain. Scale bars: 20 μm; gcl: granular cell layer; pc: purkinje cell layer; ml: molecular layer. (C) Picture of brains from a WT and a α_3_^+/D801Y^ mouse, no gross mass change of cerebellum was observed. Scale bar represent 1 mm per tick. (D) Hematoxylin and eosin staining of cerebellar slices from WT and α_3_^+/D801Y^ mice. (E) Immunofluorescent calbindin staining of Purkinje cells in cerebellar slices from WT and α_3_^+/D801Y^ mice. Number of Purkinje cells was quantified as mean number of Purkinje cells per 100 μm (N = 3 (animals), n = 6 (slices) for both WT and α_3_^+/D801Y^). Scale bar 100 μm. All data shown are means ± SEM. *p<0.05, **P<0.01.

α_3_^+/D801Y^ mice express around 80% of the α_3_ isoform protein compared to WT mice in cerebellum (p = 0.0034 for p0 and p = 0.0010 for p70, t-test), and likely as a compensation mechanism, α_1_ isoform levels are slightly higher in α_3_^+/D801Y^ mice compared to WT littermates (p = 0.0113 for p0 and p = 0.0140 for p70, t-test), both at birth and in adulthood (Fig 4A)(Full length Western blots shown in Supplementary S1 Fig). To specifically address the expression of α_1_ and α_3_ isoforms in Purkinje cells, immunofluorescence staining of cerebellar slices with antibodies against the α_1_ and α_3_ isoforms was studied (Fig 4B). WT Purkinje cells showed no expression of the α_1_ isoform, whereas strong staining of the α_3_ isoform was noted in all Purkinje cells. Notably, the same pattern of absence of expression of the α_1_ isoform was observed in Purkinje cells in the α_3_^+/D801Y^ mice, suggesting that mechanisms to compensate for loss of α_3_ isoform activity are lacking in Purkinje cells.

### Normal gross cerebellar morphology in α_3_^+/D801Y^ mice

Loss of cerebellar neurons has been reported in some patients with *ATP1A3* mutations [28, 29]. We therefore compared gross cerebellar morphology in the α_3_^+/D801Y^ mice and WT littermates. No visible loss of cerebellar mass was evident (Fig 4C) and cerebellar morphology appeared normal in stained slices from both α_3_^+/D801Y^ mice and control WT littermates (Fig 4D). Furthermore, quantification of Purkinje cells positive for calbindin revealed comparable numbers in α_3_^+/D801Y^ mice and in WT littermates (Fig 4E) (p = 0.59, t-test).

### α_3_^+/D801Y^ mice exhibit irregular cerebellar activity and abnormal high-frequency burst-like firing

**Fig 5:**
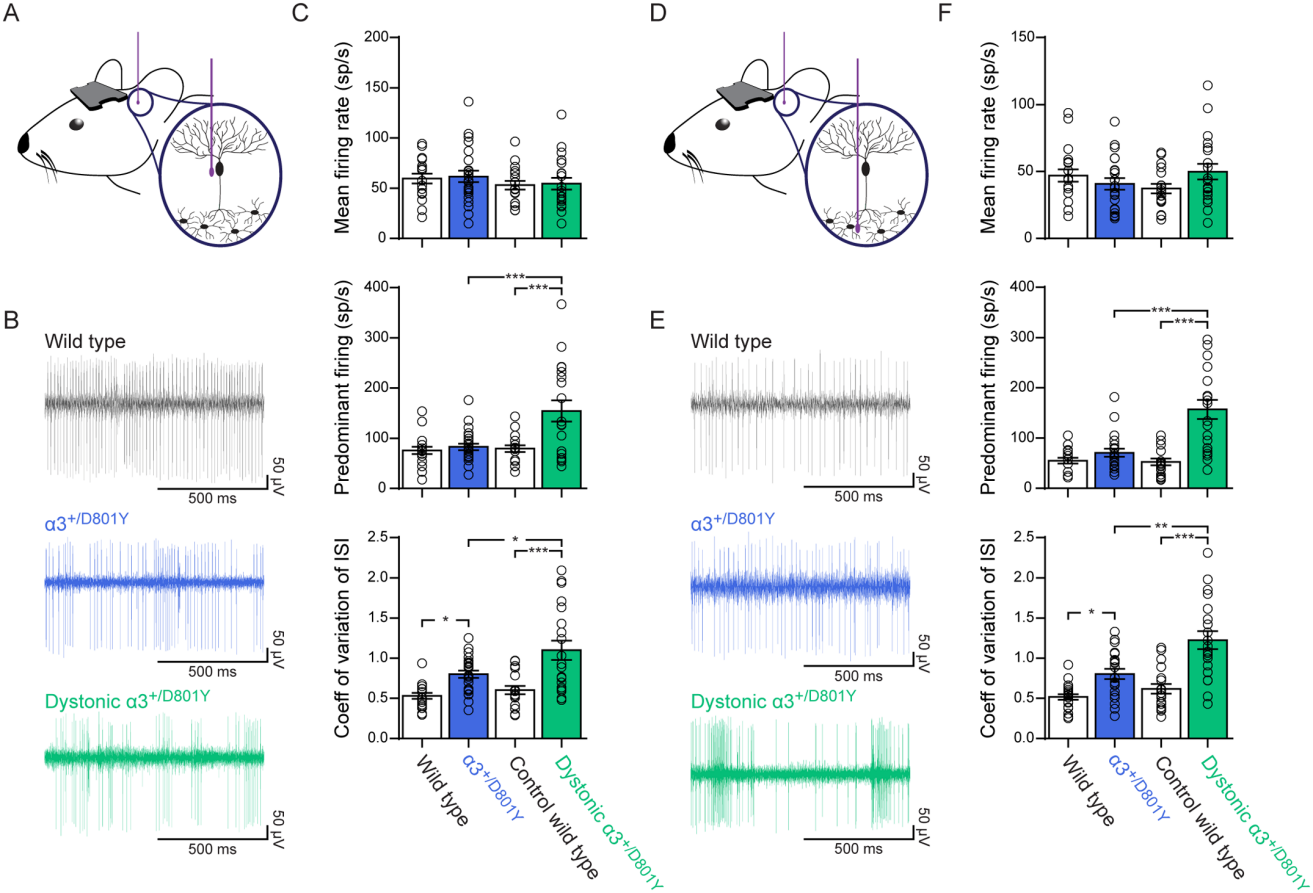
Cerebellar activity. *In vivo* recordings of awake α_3_^+/D801Y^ mice revealed irregular firing of Purkinje cells and DCN neurons, which during dystonic spells was further exacerbated and turned into periods of abnormal high-frequency bursting. (A) Illustration of an *in vivo* recording of Purkinje cells in awake head-restrained mice. (B) Representative raw traces of Purkinje cells recorded from WT, α_3_^+/D801Y^ at baseline, and α_3_^+/D801Y^ mice during dystonic attack induced by cold water. Scale bars: 500 ms by 50 μV. (C) Average firing rate (upper), predominant firing rate (middle) and CV ISI (lower) of Purkinje cells from WT (N = 4 (animals), n = 19 (cells)), α_3_^+/D801Y^ at baseline (N = 5, n = 23), control WT exposed to cold water (N = 3, n = 18) and α_3_^+/D801Y^ mice during dystonic attacks induced by cold water (N = 4, n = 20). (D) Illustration of an *in vivo* recording of DCN neurons in awake head-restrained mice. (E) Representative raw traces of DCN neurons recorded from WT, α_3_^+/D801Y^ at baseline, and α_3_^+/D801Y^ mice during dystonic attack induced by cold water. (F) Average firing rate (upper), predominant firing rate (middle) and CV ISI (lower) of DCN neurons from WT (N = 4 (animals), n = 21 (cells)), α_3_^+/D801Y^ at baseline (N = 5, n = 21), control WT mice exposed to cold water (N = 3, n = 18) and α_3_^+/D801Y^ mice during dystonic attacks induced by cold water (N = 4, n = 20). All data shown are means ± SEM. *p<0.05, **p<0.01, ***p<0.001.

Next, to investigate if cerebellar neuronal activity was affected, we performed *in vivo* single-unit extracellular recordings of Purkinje cells (Fig 5A) in awake head-restrained α_3_^+/D801Y^ mice and WT littermates (representative raw traces are in Fig 5B).

Comparing α_3_^+/D801Y^ mice with WT littermates under baseline conditions, α_3_^+/D801Y^ Purkinje cells exhibited the same mean firing rate as WT (α_3_^+/D801Y^: 62 ± 6 sp/s; WT: 60 ± 5 sp/s (p = 0.9923, two-way ANOVA with genotype (WT versus α_3_^+/D801Y^) and condition (baseline versus hypothermia) as main factors followed by Tukey's multiple comparisons test)) (Fig 5C_upper_) and their predominant firing rates were also comparable (α_3_^+/D801Y^: 83 ± 7 sp/s; WT: 76 ± 7 sp/s (p = 0.9693)) (Fig 5C_middle_). However, when investigating the regularity of the firing pattern using the coefficient of variation of interspike intervals (CV ISI), defined as the standard deviation of ISIs/mean ISI, α_3_^+/D801Y^ mice exhibited a slightly, but significantly, higher CV ISI compared to WT littermates (α_3_^+/D801Y^: 0.80 ± 0.05; WT: 0.53 ± 0.04 (p = 0.0377)) (Fig 5C_lower_). Moreover, numerous short pauses were evident in the raw recordings from α_3_^+/D801Y^ mice under baseline conditions, which contribute to the increased irregularity compared to WT (Fig 5B, blue trace).

Next we recorded from α_3_^+/D801Y^ mice undergoing dystonic attacks induced by a 4 min cold-water swim, and also from control WT littermates after identical cold-water exposure. Mean firing rate of the Purkinje cells was unaltered in dystonic α_3_^+/D801Y^ mice (dystonic α_3_^+/D801Y^: 55 ± 6 sp/s; control WT: 53 ± 4 sp/s (p = 0.9966)) (Fig 5C_upper_). However, in contrast to α_3_^+/D801Y^ mice at baseline, dystonic α_3_^+/D801Y^ mice exhibited high-frequency burst-like firing episodes of 40–80 ms in length, which occurred episodically throughout the whole duration of the induced dystonic attacks. This was evident from the raw traces (Fig 5B, green trace), as well as from the significantly higher predominant firing rate compared to control mice (dystonic α_3_^+/D801Y^: 155 ± 21 sp/s, with individual cells as high as 367 sp/s; control WT: 80 ± 7 sp/s, with individual cells as high as 144 sp/s (p = 0.0002)) and higher CV ISI (dystonic α_3_^+/D801Y^: 1.09 ± 0.11; control WT: 0.60 ± 0.05 (p<0.0001)).

The predominant firing rate and CV ISI for α_3_^+/D801Y^ mice during dystonia were also significantly higher compared to the same α_3_^+/D801Y^ mice at baseline (p = 0.0002 and p = 0.0152), whereas there was no significant difference in these parameters between WT mice exposed to the cold water (control WT) and baseline WT mice (p = 0.9959 and p = 0.8977) demonstrating that the abnormal Purkinje cell activity in dystonic α_3_^+/D801Y^ mice depends on the presence of the mutant D801Y α_3_ isoform and is not merely a response of all mice to hypothermia.

Purkinje cells form the sole output from the cerebellar cortex and make strong inhibitory synaptic connections onto the deep cerebellar nuclei (DCN) neurons, effectively modulating their activity. As the DCN provide the main cerebellar output, we next recorded DCN neurons (Fig 5D) to explore if cerebellar output was altered in α_3_^+/D801Y^ mice (representative raw traces are shown in Fig 5E)

Like the Purkinje cells, DCN neurons in α_3_^+/D801Y^ mice at baseline conditions exhibited no alteration in mean firing rate (α_3_^+/D801Y^: 41 ± 4 sp/s; WT: 47 ± 5 sp/s (p = 0.7693)) or predominant firing rate (α_3_^+/D801Y^: 71 ± 8 sp/s; WT: 55 ± 6 sp/s (p = 0.752)) (Fig 5F_upper and middle_). However, α_3_^+/D801Y^ DCN neurons did exhibit a significant higher CV ISI (α_3_^+/D801Y^: 0.81 ± 0.06; WT: 0.52 ± 0.03 (p = 0.0297)) (Fig 5F_lower_), indicating that cerebellar output was more irregular in α_3_^+/D801Y^ mice compared to WT littermates.

In dystonic α_3_^+/D801Y^ mice, DCN neurons exhibited periods with high-frequency burst-like firing similar to our observations in Purkinje cells, with the same duration and episodic nature. Their mean firing rate was comparable to that of control WT littermates (dystonic α_3_^+/D801Y^: 50 ± 6 sp/s; control WT: 37 ± 4 sp/s (p = 0.2603)) (Fig 5F_upper_), but their predominant firing rate (dystonic α_3_^+/D801Y^: 157 ± 19 sp/s with individual cells as high as 296 sp/s; control WT: 53 ± 7 sp/s with individual cells as high as 105 sp/s (p<0.0001)) and CV ISI (dystonic α_3_^+/D801Y^: 1.2 ± 0.11; control WT: 0.62 ± 0.06 (p<0.0001)) were both significantly higher than those of control WT littermates (Fig 5F_middle and lower_).

### The D-to-Y mutation impairs Na^+^/K^+^ exchange

**Fig 6:**
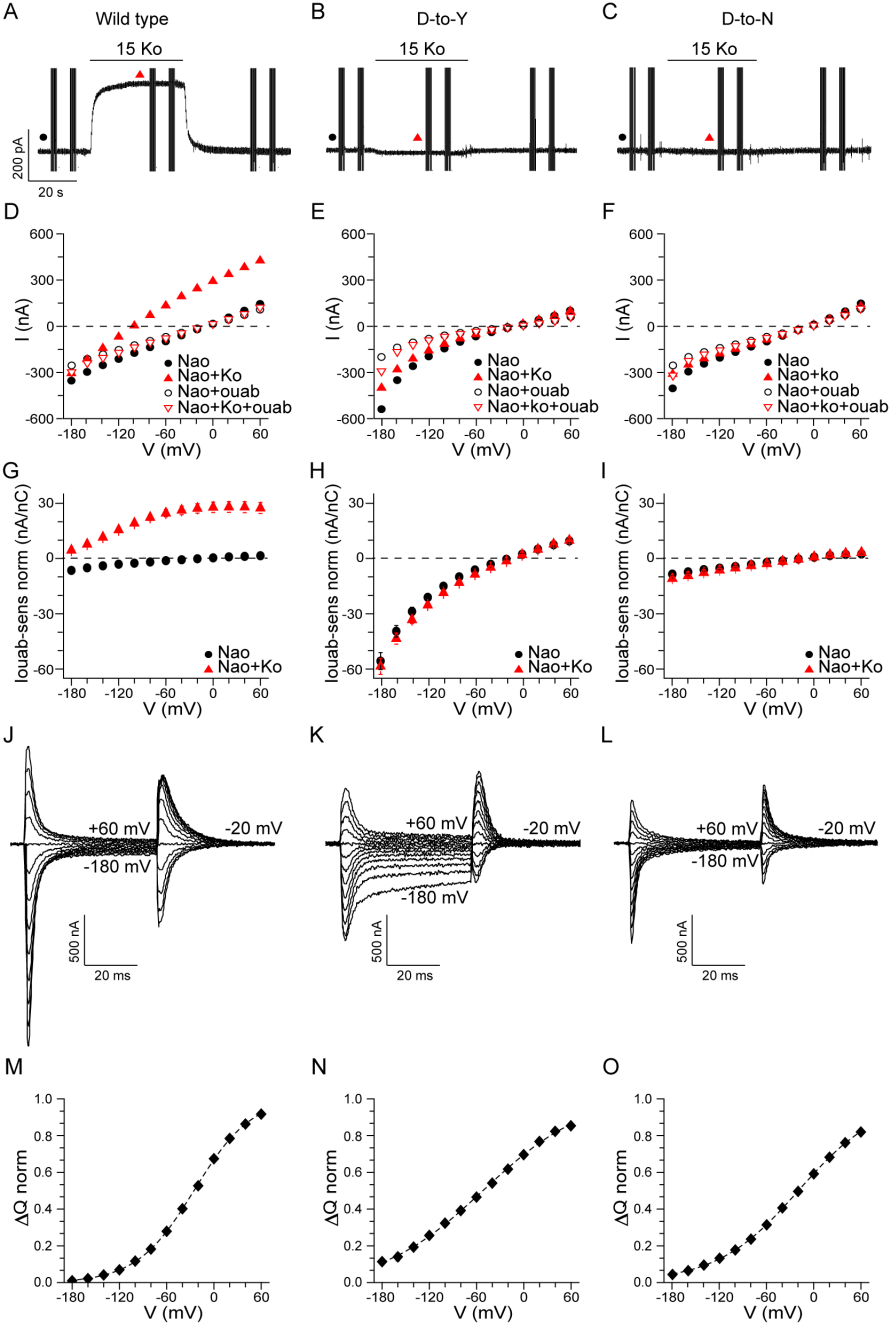
*In vitro* pump function. Functional assays of Na^+^/K^+^ ATPases with substitutions in the disease hotspot aspartate residue. (A, B, C) Currents recorded in Na^+^-loaded oocytes expressing exogenous ouabain-resistant Na^+^/K^+^-ATPases without (wild type, A), or with, a D-to-Y (B) or D-to-N (C) mutation at position 801 equivalent, held at -20 mV, exposed to 125 mM Na^+^ solution at pH 7.6 containing 1 μM ouabain (to silence endogenous Na^+^/K^+^-ATPases), with 15 mM K^+^ added as indicated by horizontal bars (Ko); the vertical lines are responses to 50-ms steps to other potentials. (D, E, F) Steady-state current levels plotted against voltage, from the recordings shown in (A, B, C) (filled symbols), in the presence (red) or absence (black) of K^+^, and from subsequent recordings in the same oocyte after inhibition of exogenously expressed pumps by 10 mM ouabain (empty symbols). (G, H, I) Average ± SEM 10 mM ouabain-sensitive steady currents (I ouab-sens) in 125 mM Na^+^, obtained by subtraction, at 0 mM K^+^ (black circle) or 15 mM K^+^ (red triangle), normalized to the maximum Na^+^ charge movement in each oocyte (J-O, below), a measure of the number of Na^+^/K^+^-ATPases; wild type (n = 4 oocytes), D-to-Y (n = 3 with K^+^, n = 6 without), D-to-N (n = 3). (J, K, L) 10 mM ouabain-sensitive pre-steady-state Na^+^ currents for wild type (J), D-to-Y (K), and D-to-N (L) Na^+^/K^+^-ATPases in 125 mM Na^+^ and 0 mM K^+^ solution obtained by subtraction of traces before and after pump inhibition; superimposed traces are from steps to voltages between -180 mV and +60 mV, and back to the holding potential, -20 mV. (M, N, O) Transient Na^+^ charge movements, ΔQ, obtained as the time integral of the transient currents at -20 mV after each voltage step, are plotted against potential during the step for wild type (M), D-to-Y (N), and D-to-N (O) Na^+^/K^+^-ATPases. Boltzmann relation fits to the ΔQ-V plots yielded maximum ΔQ values used for normalization (ΔQ norm), and mean fit values for effective valence, zq (wild type: 0.68±0.01, n = 9; D-to-Y: 0.38±0.02, n = 6; D-to-N: 0.48±0.02, n = 9), and for midpoint voltage (wild type: -24±1 mV, n = 9; D-to-Y: -51±3 mV, n = 6; D-to-N: -19±2 mV, n = 9); maximum ΔQ for D-to-Y pumps is likely underestimated due to the lower zq, so that D-to-Y currents normalized to maximum charge (H, above) may be overestimated; averaged ΔQ norm-V distributions are shown. See also Supplementary S2 Fig.

To elucidate the molecular mechanistic consequences of the RDP/AHC-causing D801Y mutation and to compare it to the AHC-causing D801N mutation, Na^+^/K^+^-ATPase-mediated currents were recorded in oocytes expressing *Xenopus laevis* orthologs of α subunit *ATP1A1*, encoding either the homologous WT aspartate, D813, or the D813Y or D813N mutation that are equivalent to WT D801, D801Y and D801N, respectively, in the human and rodent α_3_ isoform, in combination with *ATP1B3* β subunit. *Xenopus ATP1A1/ATP1B3* pumps were studied because these are believed to be the native isoforms in *Xenopus* oocytes [30, 31], an established system for high-resolution measurements of Na^+^/K^+^ pump function. Moreover, because the D801-equivalent aspartate is absolutely conserved in all Na^+^/K^+^-ATPase α subunits of all species and plays a crucial role in K^+^-ion binding, the D-to-Y and D-to-N substitutions may be expected to cause comparable disruptions of function in all Na^+^/K^+^-ATPase isoforms. Unlike WT Na^+^/K^+^ pumps (Fig 6A, 6D and 6G), neither D-to-Y (Fig 6B, 6E and 6H) nor D-to-N (Fig 6C, 6F and 6I) mutant Na^+^/K^+^-ATPases were able to generate the outward (positive) current on exposure to high external K^+^ (K^+^_o_) that signifies normal electrogenic extrusion of 3 Na^+^_i_ in exchange for import of 2 K^+^_o_ in each ATPase transport cycle. D-to-Y or D-to-N mutant Na^+^/K^+^-ATPases similarly failed to demonstrate any K^+^_o_-activated outward current when competing external Na^+^ was absent (Supplementary S2 Fig). However, in the presence of external Na^+^ but absence of K^+^_o_, thereby precluding Na^+^/K^+^ exchange even in WT Na^+^/K^+^ pumps, both D-to-Y (Fig 6K and 6N) and D-to-N (Fig 6L and 6O) Na^+^/K^+^-ATPases, like WT Na^+^/K^+^-ATPase (Fig 6J and 6M), generated robust transient currents in response to membrane potential jumps. Those ouabain-inhibited Na^+^ charge movements reveal the time course of the major conformational change of Na^+^-bound phosphorylated Na^+^/K^+^-ATPases that, in one direction encloses the three Na^+^ ions and, in the other, releases them to the cell exterior [32]. Also like WT Na^+^/K^+^-ATPases (Fig 6G and 6J), both D-to-Y (Fig 6H and 6K) and D-to-N (Fig 6I and 6L) Na^+^/K^+^-ATPases still generated the small steady inward currents at large negative potentials that reflect pump-mediated import of protons [32]; in WT, that proton current is seen clearly only without K^+^_o_ (Fig 6G), when overlapping outward Na^+^/K^+^ exchange current was absent, but in D-to-Y (Fig 6H) and D-to-N (Fig 6I), which are both incapable of generating outward Na^+^/K^+^ exchange current, that inward current was evident with or without K^+^_o_. Indeed, because these mutant Na^+^/K^+^-ATPases are incapable of tightly binding external K^+^, they are effectively permanently trapped in the phosphorylated conformations that reversibly release the three bound Na^+^ one at a time to the extracellular medium; it is precisely those conformations that carry out proton import. Importantly, not all effects of D-to-Y and D-to-N mutations were identical under all conditions, with Fig 6H and 6I (Supp. S2H and S2I Fig) suggesting that D-to-Y Na^+^/K^+^-ATPases possibly support larger proton influx than D-to-N Na^+^/K^+^-ATPases at negative resting potentials.

## Discussion

In this study, we found that the α_3_^+/D801Y^ mouse model exhibited prolonged episodes of hypothermia-induced dystonia, which usually began abruptly with hyper-extension of limbs and developed into abnormal postures and twisting movements, characteristic of dystonia [1, 2, 33]. To our knowledge, hypothermia has not previously been shown to trigger dystonia using animal models. Nevertheless, that altered temperature can induce phenotypes in the α_3_^+/D801Y^ mice is not completely unexpected, as several clinically reported triggers for RDP, AHC, and CAPOS, involve a change in body temperature [8, 10]. Prolonged exercise, alcohol consumption, and fever, all of which raise body temperature, are among the most frequently reported triggers of RDP [23]. Likewise, CAPOS can be induced by fever [9]. Furthermore, exposure to both cold and warm temperatures has been reported to trigger attacks in AHC patients [10, 15]. Nevertheless, given that hyperthermia seems to be a common trigger in *ATP1A3* patients, we also tested this in the α_3_^+/D801Y^ mice by exposing them to a heated environment that raised their body temperature to 40.4 ± 0.3°C, a physiologically relevant fever level. However, this experimentally induced hyperthermia failed to provoke any attacks in the α_3_^+/D801Y^ mice despite the facts (i) that it induced symptoms of hyperthermia, also seen in WT littermates, including immobility or circling [34], and (ii) that 10- to 14-day-old WT mice have been reported to undergo febrile seizures at body temperatures averaging 41.3°C [34].

We found that both Purkinje cells and their synaptic target, the DCN neurons, fire significantly more irregularly in the α_3_^+/D801Y^ mice compared to WT animals, a finding that correlates with observed motor deficits in the α_3_^+/D801Y^ mice, which are similarly found in all *ATP1A3*-related disorders [8]. The irregular firing was further exacerbated, and evolved into abnormal high-frequency burst-like firing, when dystonia was induced in the α_3_^+/D801Y^ mice, similar to changes in cerebellar activity noted during dystonia induced in WT mice by brain perfusion with low-dose ouabain [35, 36]. In further support of cerebellar involvement in α_3_ isoform-related dystonia, heterozygous α_3_ knock-out mice developed increased symptoms of dystonia after cerebellar perfusion with the excitatory glutamatergic agonist, kainate, and they exhibited enhanced inhibitory neurotransmission in the cerebellar cortex compared to WT mice [24]. Why exactly the cerebellum appears to be highly susceptible to dysfunction upon alterations of α_3_ isoform activity remains to be firmly established. But a likely explanation is that Purkinje cells, as also shown here, express only the α_3_ isoform of the Na^+^/K^+^ pump and not the otherwise ubiquitously expressed α_1_ isoform [24, 25], and also do not appear to be able to express the α_1_ isoform as a compensatory response to α_3_ isoform dysfunction. This special characteristic of Purkinje cells is supported by the finding that shRNA-mediated knock-down of the α_3_ isoform led to disruption of the intrinsic firing of Purkinje cells, but not of DCN neurons which express both α_1_ and α_3_ isoforms, when synaptic inputs were inhibited *in vitro* [27]. How hypothermia affects the firing of the cerebellar neurons and induces dystonia in the α_3_^+/D801Y^ mice remains to be elucidated. An earlier study found that the intrinsic activity of Purkinje neurons in culture was increasingly slowed as the cells were cooled below 20°C, with the duration of action potentials increasing as their frequency decreased [37]. This temperature range correlates with the induction of attacks in the α_3_^+/D801Y^ mice we observed when body temperature fell to 20°C. Furthermore, the spread of the ISI became larger in the cultured Purkinje cells as the temperature was lowered [37], echoing the increase in CV ISI during hypothermia-induced dystonia in the α_3_^+/D801Y^ mice. In cerebellar slices, the firing of Purkinje cells was furthermore shown to be particular affected by lowering the temperature [38], suggesting that Purkinje cells are highly temperature sensitive. Maintenance of normal intrinsic activity of Purkinje cells depends on function of their α_3_ isoform Na^+^/K^+^-ATPases [26, 27]. Although α_3_ isoform Na^+^/K^+^-ATPases in WT mice are presumably slowed by low temperature, the complete loss of Na^+^/K^+^ exchange by α_3_ isoform Na^+^/K^+^-ATPases bearing the D-to-Y mutation in cerebellar Purkinje neurons of α_3_^+/D801Y^ mice may be expected to impair their ability to adequately sustain electrical activity, compared to WT mice, at similarly low body temperatures.

Clinically, AHC and RDP have been considered to be distinct disorders, although with overlapping features [8]. The same appears true for mice, as the α_3_^+/D801Y^ mice in some aspects phenotypically differ from Mashlool (α_3_^+/D801N^) mice [39] and Myshkin (α_3_^+/I810N^) mice [40] that are heterozygous for the AHC mutations, D801N and I810N, respectively (Table 1). Both Mashlool and Myshkin mice exhibited spontaneous recurrent tonic clonic seizures [39, 40]. In contrast, although α_3_^+/D801Y^ mice have a lowered threshold for PTZ-induced seizure, they did not develop spontaneous seizures [22]. Mashlool mice, furthermore, effectively modeling AHC closely by exhibiting hemiplegic episodes of relatively long duration, but also short dystonic spells, upon water exposure [6, 39]. Here we show that α_3_^+/D801Y^ mice suffer from abrupt hypothermia-inducible dystonia that recapitulates the abrupt triggerable onset of symptoms in *ATP1A3* patients [41]. These induced, and EMG-confirmed, dystonic attacks lasted noticeably longer than those observed in Mashlool mice (α_3_^+/D801Y^: 39.2 ± 2.5 min; Mashlool (α_3_^+/D801N^): 0.07 ± 0.005 min) [39]; however, the dystonic attacks in α_3_^+/D801Y^ mice were not persistent as in RDP patients [23], and could thus resemble spells noted in AHC patients, except that the induced attacks did not include EMG hemiplegia activity or ECoG seizure activity that could be expected of such episodic spells of AHC patients [8]. This possibly reflects the intermediate nature of the D801Y mutation that has been associated with RDP and AHC [5, 11, 12].

**Table 1:** Phenotypes of α_3_ isoform knock-in mouse models. Comparison of major phenotypes of α_3_^+/D801Y^, Mashlool (α_3_^+/D801N^) and Myshkin (α_3_^+/I810N^) mice to corresponding RDP and AHC symptoms.

	Mouse model phenotypes			Clinical symptoms	
	α_3_^+/D801Y^	α_3_^+/D801N^	α_3_^+/I810N^	RDP	AHC
**Motor deficits**	Deficits in motor-related behavioural tests	Abnormal posture and gait Hind limb clasping Deficits in motor- related behavioural tests	Abnormal posture and gait Hind limb clasping Deficits in motor-related behavioural tests	Postural instability, ataxia, and bradykinesia	Ataxia, chorea, and oculomotor apraxia
**Cognitive defects**	Decreased cognitive function in spatial and fear-based memory tests	Decreased cognitive function in spatial and novel recognition memory tests	Decreased cognitive function in spatial, novel, and fear-based memory tests	Infrequent and moderate	Universal and severe
**Hemiplegia**	Not reported	Inducible. Average duration of 1.6 min	Not reported	No	Frequently recurring spells
**Dystonia**	Inducible Average duration of 39 min Pronounced co-contraction in EMG recordings	Inducible Average duration of 0.07 min	Not reported	Severe and persistent	Recurring spells
**Epileptic seizures**	No	Spontaneous	Spontaneous and inducible	Infrequent	Occurs in >50% of patients. Includes tonic, tonic-clonic, and myoclonic spells
**Triggers**	Hypothermia (dystonia)	Water exposure and handling (hemiplegia, dystonia)	Vestibular stress (seizure)	Fever, infection, alcohol consumption, physical exhaustion, emotional stress	Bathing, extreme heat or cold, visual and auditory stimuli, emotional stress
**References**	[22]	[39]	[40, 45, 46, 47]	[8, 23, 41, 48, 49]	[8, 12, 15, 50, 51]

To further explore how different mutations might cause this spectrum of neurological features, we made side-by-side comparisons of the effects of the D801Y and D801N mutations on the function of otherwise identical Na^+^/K^+^-ATPase isoforms, in the same cells and under the same conditions. We show that the D-to-Y and D-to-N mutations both abolish Na^+^/K^+^ exchange, in accordance with the known crucial role of this conserved aspartate residue to coordinate both transported K^+^ ions in the pump binding sites [19, 20], and with previous reports that the D801N mutation abolishes K^+^ occlusion [21] and K^+^-activated outward current [42], and reduced forward cycling [43]. Nevertheless, preservation of Na^+^/K^+^-ATPase-mediated transient Na^+^ charge movements in both D-to-Y and D-to-N Na^+^/K^+^-ATPases demonstrates that both mutants remain capable of binding 3 Na^+^_i_ ions, of consequent Na^+^-dependent phosphorylation, and of essential Na^+^-releasing and -rebinding conformational changes [32], as previously inferred for D801N from ouabain-binding measurements [44]. Intriguingly, the Na^+^-bound phosphorylated conformations that are shown here to be preserved in the D-to-Y and D-to-N Na^+^/K^+^-ATPases, and in which their inability to bind K^+^ ions dooms these mutants to spend most of their time, comprise precisely those conformations that support pump-mediated proton import [32]. In other words, whereas D-to-Y and D-to-N mutants must both be viewed as loss-of-function in terms of Na^+^/K^+^ exchange, they must both be considered gain-of-function in terms of their exclusive occupancy of proton-importing pump states.

As the failure of Na^+^/K^+^ exchange by the D-to-Y and D-to-N mutations demonstrated here in *Xenopus* oocytes at room temperature under optimal [K^+^]_o_, [Na^+^]_i_, and [ATP] conditions is expected to be recapitulated in D801Y and D801N α_3_ isoform Na^+^/K^+^-ATPases under physiological conditions in mice, then α_3_^+/D801Y^ mice and Mashlool (α_3_^+/D801N^) mice should both be effectively haploinsufficient in terms of α_3_ isoform Na^+^/K^+^-ATPase-mediated Na^+^ extrusion. However, we found not all effects of D-to-Y and D-to-N mutations to be identical under all conditions, suggesting that D-to-Y Na^+^/K^+^-ATPases possibly support larger proton influx than D-to-N Na^+^/K^+^-ATPases at negative resting potentials, like those of neurons. Whether phenotypical differences attributed to D801Y versus D801N mutation in α_3_ isoform pumps, such as clinical diagnoses of RDP rather than AHC in patients, and phenotypical differences between α_3_^+/D801Y^ and Mashlool mice, reflect these relatively small functional differences cannot be concluded at present, and this requires further investigation. The conclusion that both D801Y and D801N are loss-of-function mutations for Na^+^/K^+^ exchange, but are effectively gain-of-function in that the mutant pumps engage full time in proton import, possibly accounts for the scarcity and severity of these *de novo* gene alterations and the apparent lack of nonsense mutations (resulting in haploinsufficiency) in the *ATP1A3* patient group [6, 10].

If this inference is correct, it has significant implications for future therapeutic intervention in *ATP1A3*-related diseases in which α_3_ isoform haploinsufficiency may be less damaging than carrying certain missense gain-of-function mutations. Thus, strategies such as exon skipping and genome editing may be selected to eliminate the mutated disease-causing allele in cases where it is considered more deleterious than loss-of-function alleles.

## Materials & methods

### Animals

All experiments were performed on 8–16 week old α_3_^+/D801Y^ mice and WT littermates on a C57/BL6JRj (Janvier) background except the electrophysiological *in vivo* recordings, which were performed on the C57BL/6JR (Jackson laboratory) background. Mice were kept at a daily 12 hour light/dark cycle. Male and female mice were included in balanced numbers.

Experimental animal protocols performed at Aarhus University were performed according to the Danish national and Institutional regulations and approved by the Animal Experiments Inspectorate under the Danish Ministry of Justice (permit numbers 2012-15-2934-00621, 2013−15−2934−00815 and 2014−15−2934−01029). Experimental animal protocols performed at Albert Einstein College of Medicine were done according to the animal guidelines set by Einstein's Institutional Animal Care and Use Committee.

### Environmental and physiological stress conditions

Each condition was tested in at least 5 mice of each genotype and repeated 3 times per mouse. After being subjected to each condition, the mice were place on a cleared table or empty cage where they were video recorded and closely monitored by the experimenter for the occurrence of an attack. Average occurrence of attacks (%) and attack duration were subsequently calculated per mouse, per condition.

Restraining. Mice were restrained in a 60 mL falcon tube for 10 min. The back of the tube was partially closed off but allowed enough air to prevent suffocation.

Tail hanging. Mice were hung from their tail for 6 minutes.

Foot shocks. Mice were placed in a custom made plastic box with metal wiring on the bottom connected to a stimulating device. Randomly timed short shocks were given for 5 min.

Exposure to fox urine. Mice were put in a closed cage with a tube containing a tissue drenched in fox urine for 10 min.

Temperate water swim. A clear plastic box (42 x 26 x 18 cm) was filled with 35°C water to a water height that forced mice to swim without the possibility to touch the box floor. Mice were placed in the water and forced to swim for 10 min.

Chronic variable stress. The mice were subjected to one of three different stressors over the course of two weeks. Mice were suspended by their tail for 6 minutes on days 1, 6 and 9. The mice were placed in glass jars with 30°C water for 6 minutes on days 2, 8 and 11. Finally, the mice were restrained for 60 minutes using DecapiCones (BrainTree Scientific, Inc., MA, USA) on days 3, 7 and 10.

Cold water swim. A clear plastic box (42 x 26 x 18 cm) was filled with 5–10°C cold water to a water height that forced mice to swim without the possibility to touch the box floor. Mice were placed in the water and forced to swim for 4 min. Mice that showed difficulty staying afloat before the 4 min mark were immediately removed from the water. Rectal body temperatures were measured just after the animals were removed from the water.

Cold environment. Mice were placed in an empty clear plastic box and placed in a -20°C environment until they displayed attacks or their body temperature reached <20°C as measured by rectal body temperature.

Elevation of body temperature. Mice were placed in a large glass beaker placed in a 43°C warm incubator for 15 min. Rectal body temperature was measured after the animals were removed from the incubator.

### Surgery

Mice were anesthetized using isoflurane (4% in 1.5 L/min O_2_ for induction and 1–2% in 1.5 L/min O_2_ for maintenance) after which the skull was exposed, cleaned and treated with OptiBond All-In-One (Kerr Corporation; Orange, CA, USA) in order to ensure adhesion of the light cured hybrid composite Charisma (Heraeus Kulzer; Hanau, Germany) to later attach implants.

In case of preparation for ECoG recordings, four small holes (0.5 mm in diameter) were subsequently drilled for implantation of the ECoG electrodes; two above bilateral primary motor cortices (+1 mm AP and ± 1 mm ML relative to bregma) and two in the interparietal bone (-1 mm AP and ± 1 mm ML relative to lambda) to accommodate the ground and reference electrodes (Fig 2A). Teflon coated silver ball-tip electrodes (~200 μm), attached to an ECoG headmount (Pinnacle Technology; Lawrence, KS, USA), were carefully inserted into the holes and fixed in place using the hybrid composite Charisma. The rest of the skull was subsequently covered with hybrid composite Charisma to ensure insulation after which the headmount was attached to the skull.

Surgical preparation for EMG recordings was similar to the ECoG procedure. In this case coated stainless steel wires were attached to the headmount and were subcutaneously led to the hind limb. In order to avoid too much pressure on the wires at hind limb level, the wires were sutured to a patch of skin in the back and hip before attaching them to the hind limb muscles. The end of the wires were partially stripped and carefully stitched to the anterior tibialis and gastrocnemius muscles of the right hind limb. Ground and reference electrodes were made shorter than the others, partially stripped and left loose at the level of the hip.

For *in vivo* electrophysiological recordings a metal bracket was attached to the front part of the skull using light cured hybrid composite Charisma. A recording chamber was constructed on top of cerebellum with dental cement and the cavity was filled with silicone.

Following surgery all animals were given flunixin (2.5 mg/kg) and 500 μl saline, after which they were allowed at least four days to recover before being subjected to experimental procedures. All animals were monitored closely for any complications on a daily basis after surgery.

### Motor behavior tests

Mice were transferred to the test room 1 hour prior to testing for acclimation. Behavioral apparatus were cleaned between tests in 70% EtOH.

Gait analysis. Front and hind paws where painted with wet paint and the mouse was allowed to run across a long sheet of white paper. Fore base width, hind base width and stride length were subsequently estimated.

Hind limb clasping. Mice were suspended by their tails for 30 sec. Each mouse was evaluated by video analysis and given a score of either 0 (no abnormal hind limb movement) or 1 (abnormal hind limb movement) in 10 sec intervals allowing a maximum score of 3. Abnormal hind limb movement was defined as the retraction of either one or both hind limb towards the midline [45].

Balance beam. Mice were tested for 3 successive days on a 1 m long, 9 mm wide suspended wooden beam. Number of slips and the time to traverse the center 80 cm of the beam was evaluated by video analysis and calculated as the mean of three trials.

Rope climb. Mice were tested on a 40 cm long and 10 mm in diameter vertical rope. Time to climb was evaluated by video analysis and estimated as the mean of three trials.

Parallel rod floor. Mice were tested in the parallel rod floor apparatus (Stoelting Co, Wood Dale, US) for 15 min. Anymaze software (Stoelting Co, Wood Dale, US) was used to record number of slips and distance traveled.

Grip strength. A grip bar was attached to the grip strength meter (Bioseb, Vitrolles, France) to allow measurements of grip strength. A mouse was picked up by its tail and lowered until it grasped the grip bar at which point the mouse was pulled away horizontally until its grip was released. Readouts of grip strength given in grams were normalized to the body weight of the mouse and calculated as the mean of five trials.

### Western blot

Cerebellum was dissected and lysed in 10 mM Tris, 150 mM NaCl, 2 mM EDTA with 1% IGEPAL and protease inhibitor (Roche, Basel, Switzerland). Lysates were separated by SDS-PAGE and electro-blotted onto nitrocellulose membranes (Pharmacia-Amersham, Amersham, UK). Primary antibodies: anti α_1_ 1:2000 (a6f-c, Developmental Studies Hybridoma Bank, US), anti α_2_ 1:1000 (07674, EMD Millipore, US), anti α_3_ 1:1000 (06172, EMD Millipore, US), and actin 1:1000 (A2066, Sigma -Aldrich, St. louis, US) overnight at 4°C. Secondary antibodies: horseradish peroxidase-conjugated pig anti-rabbit and pig anti-mouse 1:2000 (Dako, Glostrup, Denmark). Visualization was done using a LAS 3000 imager (Fujifilm, Tokyo, Japan) with Amersham ECL Western Blotting Detection Kit (GE Healthcare, Buckinghamshire, UK) as detection reagent. Full-length Western blots are shown in Supplementary S2 Fig.

### Fluorescence immunohistochemistry

Cryo sections (15 μm thickness) were blocked in 5% donkey serum PBS/Triton X-100 0.25% for 1 hour at RT. Primary antibodies (anti α_1_ (1:400) (a6f-c, Developmental Studies Hybridoma Bank); anti α_3_ (1:300) (06172, EMD Millipore, US); anti calbindin (1:400) (ab82812, Abcam, Cambridge, UK) were applied in 1% donkey serum PBS/Triton X-100 0.25% overnight at 4°C. Secondary labelling was done with Alexa Fluor fluorescent-conjugated secondary antibodies (Alexa Fluor 488 donkey anti rabbit (A21206, Life Technologies, Carlsbad, CA, USA); Alexa Fluor 568 donkey anti mouse (A10037, Life Technologies, Carlsbad, CA, USA) (1:350) in 1% donkey serum PBS/Triton X-100 0.25% for 1 hour at RT. Hoechst (1:10000) (Life technologies, Carlsbad, CA, USA) in PBS was used to counterstain the nuclei. Sections were mounted using fluorescence mounting medium (Dako, Glostrup, Denmark) and analysed on a LSM510 laser-scanning confocal microscope using a 40x C-Apochromat water immersion objective NA 1.2 (Carl Zeiss, Göttingen, Germany). Zen 2011 software (Carl Zeiss, Göttingen, Germany) was used for analysis and image capturing.

### Functional characteristics of mutant Na^+^/K^+^-ATPases

Mutagenesis and expression. The substitution C113Y (equivalent to C101Y in human α_3_) was introduced into Xenopus Na^+^/K^+^-ATPase α_1_ subunits to render them ouabain resistant so their activity could be isolated after silencing endogenous ouabain-sensitive Xenopus Na^+^/K^+^-ATPases by maintained exposure to 1 μM ouabain. C113Y α_1_ subunits (here designated “wild type”) then served as templates for D813Y and D813N mutations (equivalent to D801Y and D801N, respectively, in human α_3_); these cysteine and aspartate residues are conserved in all Na^+^/K^+^-ATPase α isoforms of all species. All substitutions were by QuikChange (Stratagene, California, US) and were verified by sequencing. cDNAs were transcribed in vitro, and 15–45 ng of Na^+^/K^+^-ATPase α_1_ subunit cRNA was coinjected with 5–15 ng of wild-type Xenopus β_3_ subunit cRNA into defolliculated Xenopus laevis oocytes, which were incubated at 18°C for 3 days before recording.

Solutions and Na^+^/K^+^-ATPase electrophysiology. External solutions contained 125 mM NaOH or tetramethylammonium (TMA)-OH, 120 mM sulfamic acid, 0 or 15 mM K^+^-sulfamate, 5 mM BaCl_2_, 1 mM MgCl_2_, 0.5 mM CaCl_2_, 10 mM Hepes (pH 7.6), plus 1 μM ouabain to inhibit native Na^+^/K^+^-ATPases; osmolality was 250–260 mosmol/Kg. To inhibit C113Y-containing ouabain-resistant Na^+^/K^+^-ATPases, 10 mM ouabain was directly dissolved into appropriate external solutions. Before recording, [Na^+^]i was raised by incubating oocytes for ≥2 h in K^+^- and Ca_2_^+^-free solution, containing 95 mM NaOH, 90 mM sulfamic acid, 10 mM TEACl, 0.1 mM EGTA, 5 mM HEPES (pH 7.6); osmolality ~210 mosmol/Kg. Two-microelectrode voltage clamp was used to record currents at 22–24°C in oocytes expressing ouabain-resistant Na^+^/K^+^-ATPases, as previously described [30]. Whole oocyte currents were acquired with an OC-725A amplifier (Warner Instruments, US), filtered at 1 kHz, and sampled at 5 kHz with an 18-bit ITC-18 A/D-D/A board controlled by Patch Master 2.20 software (Instrutech; HEKA, US). Currents at potentials between -180 and +60 mV, in 20 mV increments, were elicited by 50-ms voltage steps from the -20 mV holding potential; steady-state currents were determined by averaging over the last 10 ms. Currents generated by ouabain-resistant Na^+^/K^+^-ATPases were obtained by subtracting current traces recorded in 10 mM ouabain from those recorded in 1 μM ouabain. Na^+^ charge movement quantities, ΔQ, were obtained as integrals of the 10 mM ouabain-sensitive transient currents at -20 mV upon termination of each voltage step in 125 mM Na^+^o, 0 mM K^+^o solution. Data was analyzed with IgorPro 6 (WaveMetric, US) and Origin 7.0 (Origin Laboratory, US).

### ECoG and EMG recordings

Recordings were performed in awake, freely moving animals using a Pinnacle data acquisition system (Pinnacle Technology; Lawrence, KS, USA). They were given 1–2 accommodation sessions to allow them to get used to the experimental setup after which experiments started. 10–30 min of baseline activity was recorded in each mouse before being subjected to a cold-water swim. ECoG or EMG activity was subsequently recorded during the attack that followed until they were fully recovered (20–50 min later). As a control, tonic-clonic seizures were induced using a Lithium Pilocarpine protocol in the same mice. 18–24 hrs prior to seizure induction, mice were given an IP injection of lithium chloride (3 mmol/kg; Sigma-Aldrich; St. Louis, MO, USA) dissolved in 0.9M NaCl after which 60–120 mg/kg of pilocarpine hydrochloride (dissolved in 0.9M saline; Sigma-Aldrich) was given IP to induce the epileptic seizure. Data were sampled at 400 Hz for ECoG and 600 Hz for EMG recordings and filtered online using a 1–150 Hz (ECoG) or 1–250 Hz (EMG) band pass filter. Signals were visualized and power spectra and cross-correlations were calculated using a custom written LabView algorithm (National Instruments; Austin, TX, USA).

### *In vivo* electrophysiology

A craniotomy was made over the cerebellum and the mice were head restrained via the implanted metal bracket in the recording setup and given one hour to acclimate before recording was initiated.

In order to record from α_3_^+/D801Y^ mice during dystonic attacks, mice were cooled in 6–8°C cold water for 4 min and immediately head restrained and after which the recording was initiated. As a control, the same experiments were done in wild type littermates.

Single-cell activity of Purkinje cells and DCN neurons was recorded extracellularly using a tungsten electrode (Thomas Recording, 2–3MΩ), which was advanced into the cerebellum until either the Purkinje cell layers or DCNs were reached (All coordinates given from Bregma: Caudal 6.45–6.55 mm, Lateral ±1.2 mm, Ventral 1.4–2.2 mm for Purkinje cell layers and 3–3.5 mm for DCN). The constructed recording chamber was filled with saline and used for ground connection. Signals were band-pass filtered (200 Hz–20 kHz) and amplified (2000×) using a custom built amplifier and then digitized (20 kHz) using a National Instruments PCI MIO 16 XEI (National Instruments Corporation, Austin, US).

Waveforms were sorted offline using characteristics of the spikes such as amplitude and energy and by principal component analysis (Offline Sorter, Plexon, US). Purkinje cells and DCN neurons were identified by location, characteristic firing rate, and the presence of complex spikes for Purkinje cells.

Average firing rate (spikes per sec, sp/s), predominant (mode) firing rate (sp/s), and coefficient of variation of interspike intervals (CV ISI) were calculated using custom made MATLAB software.

### Statistical analysis

Statistical analyses were performed in Graphpad Prism software (GraphPad Software Inc, La Jolla, US). Student’s t-test (unpaired or paired) was used to determine significance when comparing two groups. Two-way ANOVAs followed by Tukey's multiple comparisons test were used to find statistically significant differences between three or more groups. Statistical analyses for each experiment are indicated in the result section with corresponding p-values. Differences were considered significant if p<0.05.

## Supporting information

S1 Fig Full length Western blots. Membranes cut at ~ 50 kDa, top part incubated with anti α1, α2 or α3 and lower part incubated with anti-actin as loading control.
(TIF)

S2 Fig *In vitro* pump function. Functional assays of wild type Na^+^/K^+^ pumps and of pumps with substitutions at the 801-position aspartate in the absence of external Na. (A, B and C) Currents recorded in Na^+^-loaded oocytes expressing exogenous ouabain-resistant Na^+^/K^+^-ATPases without (wild type, A), or with, a D-to-Y (B) or D-to-N (C) mutation at position 801 equivalent, held at -20 mV, exposed to Na^+^-free solution at pH 7.6 containing 1 μM ouabain (to silence endogenous pumps), with 15 mM K^+^ added as indicated by horizontal bars (Ko); the vertical lines are responses to 50-ms steps to other potentials. These recordings are from the same experiments as in Fig 6A, 6B and 6C. (D,E and F) Steady-state current levels plotted against voltage, from the recordings shown in A,B and C (filled symbols), in the presence (red) or absence (black) of K^+^, and from subsequent recordings in the same oocyte after inhibition of exogenously expressed pumps by 10 mM ouabain (empty symbols). (G,H and I) Average ± SEM 10 mM ouabain-sensitive steady currents (I ouab-sens), obtained by subtraction, at 0 mM K^+^ (black circle) or 15 mM K^+^ (red triangle), normalized to the maximum Na^+^ charge movement, a measure of Na^+^/K^+^-ATPase number, determined for each oocyte from transient currents in 125 mM Na^+^ and 0 mM K^+^; wild type (n = 4 oocytes), D-to-Y (n = 5 with K+, 6 without), D-to-N (n = 3); at -180 mV, D-to-Y currents averaged -120 nA/nC in 0 mM Na^+^ with 0 mM K^+^, and -300 nA/nC in 0 mM Na^+^ with 15 mM K^+^.
(TIF)

S1 Video Cold water-induced attacks. (00:02–00:20) Example of dystonia-like postures in a α_3_^+/D801Y^ mouse with a WT littermate showing no abnormal postures. (00:20–01:45) Example of dystonia-like postures with hindlimbs severely hyperextended caudally that develop into convulsions with abnormal postures and twisting movement in a α_3_^+/D801Y^ mouse (second from left) with WT littermates as controls. All videos were captured after cold-water swim for 4 min and are representative for the attacks observed in α_3_^+/D801Y^ mice after hypothermia.
(MP4)

S2 Video Balance beam performance. (00:02–00:05) WT mouse crossing the beam representative of an average performance. (00:05–00:15) α_3_^+/D801Y^ mouse crossing the beam representative of an average performance. (00:15–00:37) α_3_^+/D801Y^ mice occasionally failed to cross the beam walking, and instead clasped the beam with their hind limbs and used their tail as support while dragging themselves forward using only their forelimbs.
(MP4)

## References

[1] Albanese A, Bhatia K, Bressman SB, Delong MR, Fahn S, Fung VS, et al Phenomenology and classification of dystonia: a consensus update. Movement disorders: official journal of the Movement Disorder Society. 2013;28(7):863–73. 23649720 10.1002/mds.25475 PMC3729880

[2] Fahn S. Concept and classification of dystonia. Advances in neurology. 1988;50:1–8. 3041755

[3] Breakefield XO, Blood AJ, Li Y, Hallett M, Hanson PI, Standaert DG. The pathophysiological basis of dystonias. Nature reviews Neuroscience. 2008;9(3):222–34. 10.1038/nrn2337 18285800

[4] Lohmann K, Klein C. Genetics of dystonia: what's known? What's new? What's next? Movement disorders: official journal of the Movement Disorder Society. 2013;28(7):899–905. 23893446 10.1002/mds.25536

[5] de Carvalho Aguiar P, Sweadner KJ, Penniston JT, Zaremba J, Liu L, Caton M, et al Mutations in the Na+/K+ -ATPase alpha3 gene ATP1A3 are associated with rapid-onset dystonia parkinsonism. Neuron. 2004;43(2):169–75. 10.1016/j.neuron.2004.06.028 15260953

[6] Heinzen EL, Swoboda KJ, Hitomi Y, Gurrieri F, Nicole S, de Vries B, et al De novo mutations in ATP1A3 cause alternating hemiplegia of childhood. Nat Genet. 2012;44(9):1030–4. 10.1038/ng.2358 22842232 PMC3442240

[7] Rosewich H, Baethmann M, Ohlenbusch A, Gartner J, Brockmann K. A novel ATP1A3 mutation with unique clinical presentation. J Neurol Sci. 2014;341(1–2):133–5. 10.1016/j.jns.2014.03.034 24713507

[8] Sweney MT, Newcomb TM, Swoboda KJ. The expanding spectrum of neurological phenotypes in children with ATP1A3 mutations, Alternating Hemiplegia of Childhood, Rapid-onset Dystonia-Parkinsonism, CAPOS and beyond. Pediatric neurology. 2015;52(1):56–64. 10.1016/j.pediatrneurol.2014.09.015 25447930 PMC4352574

[9] Demos MK, van Karnebeek CD, Ross CJ, Adam S, Shen Y, Zhan SH, et al A novel recurrent mutation in ATP1A3 causes CAPOS syndrome. Orphanet J Rare Dis. 2014;9:15 10.1186/1750-1172-9-15 24468074 PMC3937150

[10] Heinzen EL, Arzimanoglou A, Brashear A, Clapcote SJ, Gurrieri F, Goldstein DB, et al Distinct neurological disorders with ATP1A3 mutations. Lancet Neurol. 2014;13(5):503–14. 10.1016/S1474-4422(14)70011-0 24739246 PMC4238309

[11] Brashear A, DeLeon D, Bressman SB, Thyagarajan D, Farlow MR, Dobyns WB. Rapid-onset dystonia-parkinsonism in a second family. Neurology. 1997;48(4):1066–9. 9109901 10.1212/wnl.48.4.1066

[12] Viollet L, Glusman G, Murphy KJ, Newcomb TM, Reyna SP, Sweney M, et al Alternating Hemiplegia of Childhood: Retrospective Genetic Study and Genotype-Phenotype Correlations in 187 Subjects from the US AHCF Registry. PloS one. 2015;10(5):e0127045 10.1371/journal.pone.0127045 25996915 PMC4440742

[13] Ishii A, Saito Y, Mitsui J, Ishiura H, Yoshimura J, Arai H, et al Identification of ATP1A3 mutations by exome sequencing as the cause of alternating hemiplegia of childhood in Japanese patients. PLoS One. 2013;8(2):e56120 10.1371/journal.pone.0056120 23409136 PMC3568031

[14] Rosewich H, Thiele H, Ohlenbusch A, Maschke U, Altmuller J, Frommolt P, et al Heterozygous de-novo mutations in ATP1A3 in patients with alternating hemiplegia of childhood: a whole-exome sequencing gene-identification study. Lancet Neurol. 2012;11(9):764–73. 10.1016/S1474-4422(12)70182-5 22850527

[15] Hoei-Hansen CE, Dali CI, Lyngbye TJ, Duno M, Uldall P. Alternating hemiplegia of childhood in Denmark: clinical manifestations and ATP1A3 mutation status. Eur J Paediatr Neurol. 2014;18(1):50–4. 10.1016/j.ejpn.2013.08.007 24100174

[16] Panagiotakaki E, De Grandis E, Stagnaro M, Heinzen EL, Fons C, Sisodiya S, et al Clinical profile of patients with ATP1A3 mutations in Alternating Hemiplegia of Childhood-a study of 155 patients. Orphanet J Rare Dis. 2015;10:123 10.1186/s13023-015-0335-5 26410222 PMC4583741

[17] Shinoda T, Ogawa H, Cornelius F, Toyoshima C. Crystal structure of the sodium-potassium pump at 2.4 A resolution. Nature. 2009;459(7245):446–50. 10.1038/nature07939 19458722

[18] Morth JP, Pedersen BP, Toustrup-Jensen MS, Sorensen TL, Petersen J, Andersen JP, et al Crystal structure of the sodium-potassium pump. Nature. 2007;450(7172):1043–9. 10.1038/nature06419 18075585

[19] Kanai R, Ogawa H, Vilsen B, Cornelius F, Toyoshima C. Crystal structure of a Na+-bound Na+,K+-ATPase preceding the E1P state. Nature. 2013;502(7470):201–6. 10.1038/nature12578 24089211

[20] Nyblom M, Poulsen H, Gourdon P, Reinhard L, Andersson M, Lindahl E, et al Crystal structure of Na+, K(+)-ATPase in the Na(+)-bound state. Science. 2013;342(6154):123–7. 10.1126/science.1243352 24051246

[21] Nielsen JM, Pedersen PA, Karlish SJ, Jorgensen PL. Importance of intramembrane carboxylic acids for occlusion of K+ ions at equilibrium in renal Na,K-ATPase. Biochemistry. 1998;37(7):1961–8. 10.1021/bi972524q 9485323

[22] Holm TH, Isaksen TJ, Glerup S, Heuck A, Bøttger P, Füchtbauer E-M, et al Cognitive deficits caused by a disease-mutation in the α3 Na+/K+-ATPase isoform. Sci Rep. 2016;6:31972 27549929 10.1038/srep31972 PMC4994072

[23] Brashear A, Dobyns WB, de Carvalho Aguiar P, Borg M, Frijns CJ, Gollamudi S, et al The phenotypic spectrum of rapid-onset dystonia-parkinsonism (RDP) and mutations in the ATP1A3 gene. Brain: a journal of neurology. 2007;130(Pt 3):828–35. 17282997 10.1093/brain/awl340

[24] Ikeda K, Satake S, Onaka T, Sugimoto H, Takeda N, Imoto K, et al Enhanced inhibitory neurotransmission in the cerebellar cortex of Atp1a3-deficient heterozygous mice. The Journal of physiology. 2013;591(13):3433–49. 10.1113/jphysiol.2012.247817 23652595 PMC3717237

[25] Peng L, Martin-Vasallo P, Sweadner KJ. Isoforms of Na,K-ATPase alpha and beta subunits in the rat cerebellum and in granule cell cultures. The Journal of neuroscience: the official journal of the Society for Neuroscience. 1997;17(10):3488–502. 9133374 10.1523/JNEUROSCI.17-10-03488.1997 PMC6573685

[26] Forrest MD, Wall MJ, Press DA, Feng J. The sodium-potassium pump controls the intrinsic firing of the cerebellar Purkinje neuron. PloS one. 2012;7(12):e51169 10.1371/journal.pone.0051169 23284664 PMC3527461

[27] Fremont R, Tewari A, Khodakhah K. Aberrant Purkinje cell activity is the cause of dystonia in a shRNA-based mouse model of Rapid Onset Dystonia-Parkinsonism. Neurobiology of disease. 2015;82:200–12. 10.1016/j.nbd.2015.06.004 26093171 PMC4641034

[28] Oblak AL, Hagen MC, Sweadner KJ, Haq I, Whitlow CT, Maldjian JA, et al Rapid-onset dystonia-parkinsonism associated with the I758S mutation of the ATP1A3 gene: a neuropathologic and neuroanatomical study of four siblings. Acta Neuropathol. 2014;128(1):81–98. 10.1007/s00401-014-1279-x 24803225 PMC4059967

[29] Sweadner KJ, Toro C, Whitlow CT, Snively BM, Cook JF, Ozelius LJ, et al ATP1A3 Mutation in Adult Rapid-Onset Ataxia. PloS one. 2016;11(3):e0151429 10.1371/journal.pone.0151429 26990090 PMC4798776

[30] Verrey F, Kairouz P, Schaerer E, Fuentes P, Geering K, Rossier BC, et al Primary sequence of Xenopus laevis Na+-K+-ATPase and its localization in A6 kidney cells. Am J Physiol. 1989;256(6 Pt 2):F1034–43. 2544104 10.1152/ajprenal.1989.256.6.F1034

[31] Good PJ, Richter K, Dawid IB. A nervous system-specific isotype of the beta subunit of Na+,K(+)-ATPase expressed during early development of Xenopus laevis. Proc Natl Acad Sci U S A. 1990;87(23):9088–92. 2174552 10.1073/pnas.87.23.9088 PMC55109

[32] Vedovato N, Gadsby DC. Route, mechanism, and implications of proton import during Na+/K+ exchange by native Na+/K+-ATPase pumps. The Journal of general physiology. 2014;143(4):449–64. 10.1085/jgp.201311148 24688018 PMC3971657

[33] Richter F, Richter A. Genetic animal models of dystonia: common features and diversities. Progress in neurobiology. 2014;121:91–113. 10.1016/j.pneurobio.2014.07.002 25034123

[34] van Gassen KL, Hessel EV, Ramakers GM, Notenboom RG, Wolterink-Donselaar IG, Brakkee JH, et al Characterization of febrile seizures and febrile seizure susceptibility in mouse inbred strains. Genes Brain Behav. 2008;7(5):578–86. 10.1111/j.1601-183X.2008.00393.x 18363854

[35] Fremont R, Calderon DP, Maleki S, Khodakhah K. Abnormal high-frequency burst firing of cerebellar neurons in rapid-onset dystonia-parkinsonism. The Journal of neuroscience: the official journal of the Society for Neuroscience. 2014;34(35):11723–32. 25164667 10.1523/JNEUROSCI.1409-14.2014 PMC4145175

[36] Calderon DP, Fremont R, Kraenzlin F, Khodakhah K. The neural substrates of rapid-onset Dystonia-Parkinsonism. Nature neuroscience. 2011;14(3):357–65. 10.1038/nn.2753 21297628 PMC3430603

[37] Gahwiler BH, Mamoon AM, Schlapfer WT, Tobias CA. Effects of temperature on spontaneous bioelectric activity of cultured nerve cells. Brain research. 1972;40(2):527–33. 5027177 10.1016/0006-8993(72)90157-6

[38] Womack M, Khodakhah K. Active contribution of dendrites to the tonic and trimodal patterns of activity in cerebellar Purkinje neurons. J Neurosci. 2002;22(24):10603–12. 12486152 10.1523/JNEUROSCI.22-24-10603.2002 PMC6758439

[39] Hunanyan AS, Fainberg NA, Linabarger M, Arehart E, Leonard AS, Adil SM, et al Knock-in mouse model of alternating hemiplegia of childhood: behavioral and electrophysiologic characterization. Epilepsia. 2015;56(1):82–93. 10.1111/epi.12878 25523819

[40] Clapcote SJ, Duffy S, Xie G, Kirshenbaum G, Bechard AR, Rodacker Schack V, et al Mutation I810N in the alpha3 isoform of Na+,K+-ATPase causes impairments in the sodium pump and hyperexcitability in the CNS. Proceedings of the National Academy of Sciences of the United States of America. 2009;106(33):14085–90. 10.1073/pnas.0904817106 19666602 PMC2729024

[41] Brashear A, Farlow MR, Butler IJ, Kasarskis EJ, Dobyns WB. Variable phenotype of rapid-onset dystonia-parkinsonism. Movement disorders: official journal of the Movement Disorder Society. 1996;11(2):151–6. 8684384 10.1002/mds.870110206

[42] Koenderink JB, Geibel S, Grabsch E, De Pont JJ, Bamberg E, Friedrich T. Electrophysiological analysis of the mutated Na,K-ATPase cation binding pocket. The Journal of biological chemistry. 2003;278(51):51213–22. 10.1074/jbc.M306384200 14532287

[43] Li M, Jazayeri D, Corry B, McSweeney KM, Heinzen EL, Goldstein DB, et al A functional correlate of severity in alternating hemiplegia of childhood. Neurobiol Dis. 2015;77:88–93. 10.1016/j.nbd.2015.02.002 25681536

[44] Kuntzweiler TA, Arguello JM, Lingrel JB. Asp804 and Asp808 in the transmembrane domain of the Na,K-ATPase alpha subunit are cation coordinating residues. J Biol Chem. 1996;271(47):29682–7. 8939901 10.1074/jbc.271.47.29682

[45] Kirshenbaum GS, Dawson N, Mullins JG, Johnston TH, Drinkhill MJ, Edwards IJ, et al Alternating hemiplegia of childhood-related neural and behavioural phenotypes in Na+,K+-ATPase alpha3 missense mutant mice. PloS one. 2013;8(3):e60141 10.1371/journal.pone.0060141 23527305 PMC3603922

[46] Kirshenbaum GS, Clapcote SJ, Duffy S, Burgess CR, Petersen J, Jarowek KJ, et al Mania-like behavior induced by genetic dysfunction of the neuron-specific Na+,K+-ATPase alpha3 sodium pump. Proc Natl Acad Sci U S A. 2011;108(44):18144–9. 10.1073/pnas.1108416108 22025725 PMC3207708

[47] Kirshenbaum GS, Dachtler J, Roder JC, Clapcote SJ. Characterization of cognitive deficits in mice with an alternating hemiplegia-linked mutation. Behavioral neuroscience. 2015;129(6):822–31. 10.1037/bne0000097 26501181 PMC4655871

[48] Dobyns WB, Ozelius LJ, Kramer PL, Brashear A, Farlow MR, Perry TR, et al Rapid-onset dystonia-parkinsonism. Neurology. 1993;43(12):2596–602. 8255463 10.1212/wnl.43.12.2596

[49] Cook JF, Hill DF, Snively BM, Boggs N, Suerken CK, Haq I, et al Cognitive impairment in rapid-onset dystonia-parkinsonism. Mov Disord. 2014;29(3):344–50. 10.1002/mds.25790 24436111 PMC3960305

[50] Verret S, Steele JC. Alternating hemiplegia in childhood: a report of eight patients with complicated migraine beginning in infancy. Pediatrics. 1971;47(4):675–80. 5089756

[51] Mikati MA, Kramer U, Zupanc ML, Shanahan RJ. Alternating hemiplegia of childhood: clinical manifestations and long-term outcome. Pediatr Neurol. 2000;23(2):134–41. 11020638 10.1016/s0887-8994(00)00157-0

