# Supplementary figures and images for: Hypothermia-induced dystonia and abnormal cerebellar activity in a mouse model with a single disease-mutation in the sodium-potassium pump
Source: PLoS Genet. 2017 May 4;13(5):e1006763. doi: 10.1371/journal.pgen.1006763 (PMC5436892; doi:10.1371/journal.pgen.1006763)

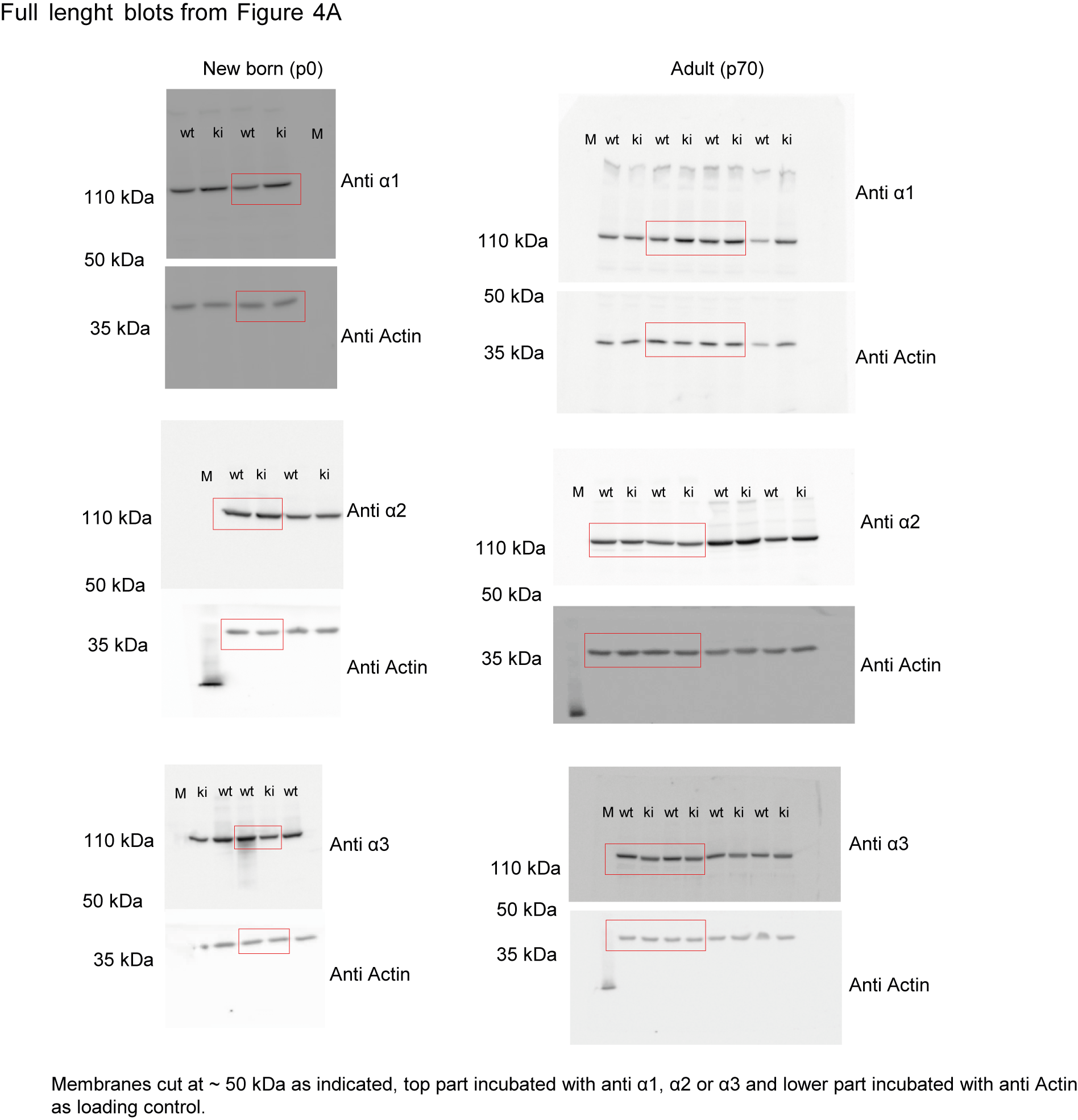

Supplement: S1 Fig — Membranes cut at ~ 50 kDa, top part incubated with anti α1, α2 or α3 and lower part incubated with anti-actin as loading control. (TIF) [file pgen.1006763.s001.tif]

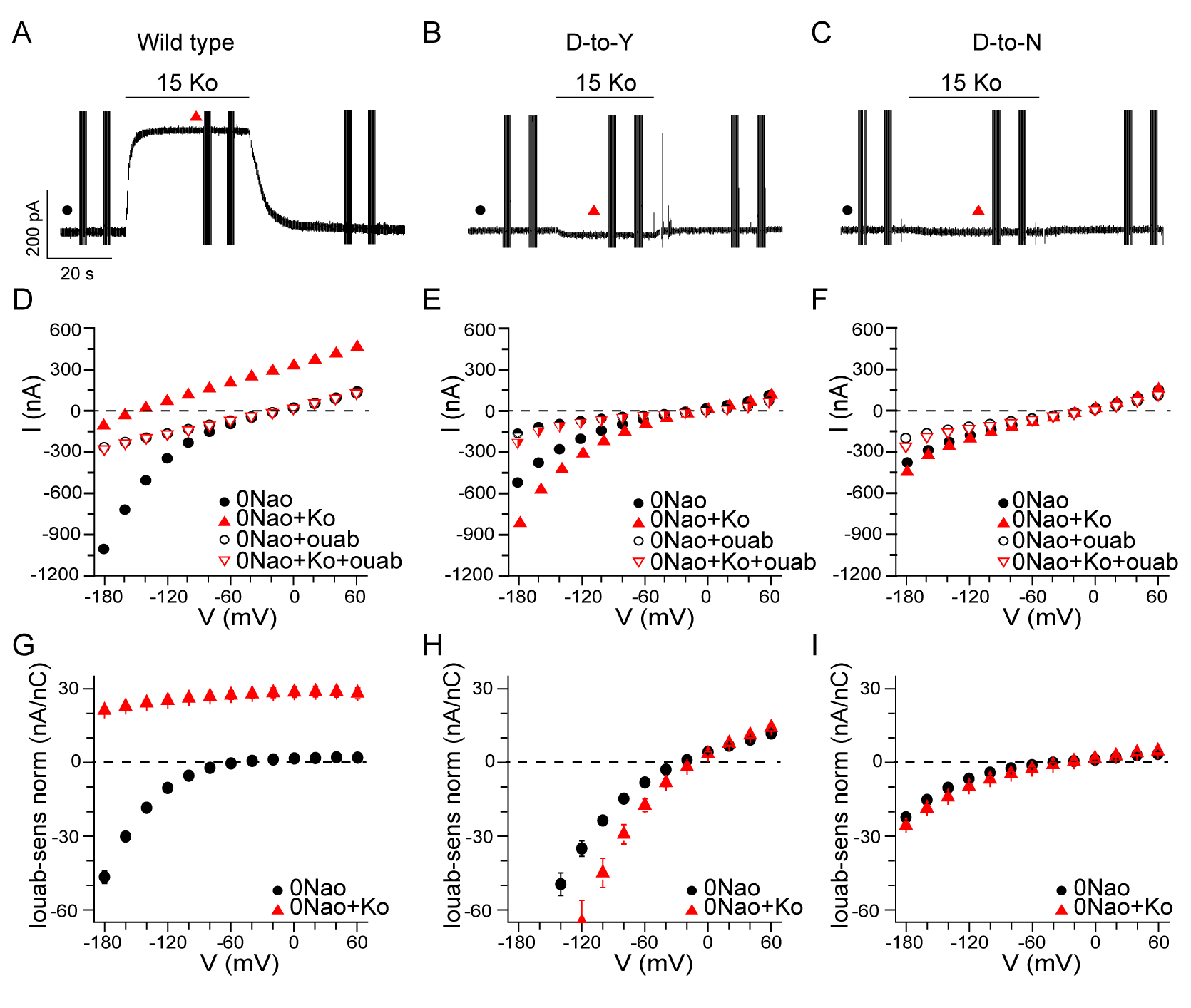

Supplement: S2 Fig — (A, B and C) Currents recorded in Na+-loaded oocytes expressing exogenous ouabain-resistant Na+/K+-ATPases without (wild type, A), or with, a D-to-Y (B) or D-to-N (C) mutation at position 801 equivalent, held at -20 mV, exposed to Na+-free solution at pH 7.6 containing 1 μM ouabain (to silence endogenous pumps), with 15 mM K+ added as indicated by horizontal bars (Ko); the vertical lines are responses to 50-ms steps to other potentials. These recordings are from the same experiments as in Fig 6A, 6B and 6C. (D,E and F) Steady-state current levels plotted against voltage, from the recordings shown in A,B and C (filled symbols), in the presence (red) or absence (black) of K+, and from subsequent recordings in the same oocyte after inhibition of exogenously expressed pumps by 10 mM ouabain (empty symbols). (G,H and I) Average ± SEM 10 mM ouabain-sensitive steady currents (I ouab-sens), obtained by subtraction, at 0 mM K+ (black circle) or 15 mM K+ (red triangle), normalized to the maximum Na+ charge movement, a measure of Na+/K+-ATPase number, determined for each oocyte from transient currents in 125 mM Na+ and 0 mM K+; wild type (n = 4 oocytes), D-to-Y (n = 5 with K+, 6 without), D-to-N (n = 3); at -180 mV, D-to-Y currents averaged -120 nA/nC in 0 mM Na+ with 0 mM K+, and -300 nA/nC in 0 mM Na+ with 15 mM K+. (TIF) [file pgen.1006763.s002.tif]
